# Effects of Weaning on Intestinal Upper Villus Epithelial Cells of Piglets

**DOI:** 10.1371/journal.pone.0150216

**Published:** 2016-03-29

**Authors:** Huansheng Yang, Xia Xiong, Xiaocheng Wang, Bie Tan, Tiejun Li, Yulong Yin

**Affiliations:** 1 Chinese Academy of Science, Institute of Subtropical Agriculture, Research Center for Healthy Breeding of Livestock and Poultry, Hunan Engineering and Research Center of Animal and Poultry Science and Key Laboratory for Agroecological Processes in Subtropical Region, Scientific Observation and Experimental Station of Animal Nutrition and Feed Science in South-Central, Ministry of Agriculture, Hunan, China; 2 School of Life Sciences, Hunan Normal University, Changsha, China; 3 Fujian Aonong Biotechnology Corporation, Xiamen, Fujian, China; Cincinnati Children's Hospital Medical Center, UNITED STATES

## Abstract

The intestinal upper villus epithelial cells represent the differentiated epithelial cells and play key role in digesting and absorbing lumenal nutrients. Weaning stress commonly results in a decrease in villus height and intestinal dysfunction in piglets. However, no study have been conducted to test the effects of weaning on the physiology and functions of upper villus epithelial cells. A total of 40 piglets from 8 litters were weaned at 14 days of age and one piglet from each litter was killed at 0 d (w0d), 1 d (w1d), 3 d (w3d), 5 d (w5d), and 7 d (w7d) after weaning, respectively. The upper villus epithelial cells in mid-jejunum were isolated using the distended intestinal sac method. The expression of proteins in upper villus epithelial cells was analyzed using the isobaric tags for relative and absolute quantification or Western blotting. The expression of proteins involved in energy metabolism, Golgi vesicle transport, protein amino acid glycosylation, secretion by cell, transmembrane transport, ion transport, nucleotide catabolic process, translational initiation, and epithelial cell differentiation and apoptosis, was mainly reduced during the post-weaning period, and these processes may be regulated by mTOR signaling pathway. These results indicated that weaning inhibited various cellular processes in jejunal upper villus epithelial cells, and provided potential new directions for exploring the effects of weaning on the functions of intestine and improving intestinal functions in weaning piglets.

## Introduction

Weaning is one of the most stressful events the pigs encounter in swine production because the weaning pigs must rapidly adapt to great changes in thediets, physical environments, and socialenvironments [1, 2]. The combined effects of these stressors changed the gastrointestinal conditions and adversely affected the health and welfare of post-weaning piglets [3]. This is especially critical in modern swine production systems, in which piglets are weaned at around 21 d of age or less. Early weaning resulted in villous atrophy and a sustained impairment of intestinal barrier function [2, 4], disturbed absorptive-secretory electrolyte and fluid balances [5–7], decreased enzymatic activities [4, 8], induced the expression of proinflammatory cytokine [9, 10], and lowered the levels of mucins [11].

The intestinal epithelium is made up of a monolayer of epithelial cells, which perform the primary functions in digesting and absorbing nutrients, and form a barrier against luminal pathogens and toxic substances [12]. The small intestinal epithelium can be divided into crypt and villi [12, 13]. The epithelial cells undergo continual renewal that involves highly coordinated processes of cellular proliferation, differentiation, and apoptosis along the crypt-villus axis (CVA). The mature epithelial cells covered villi arise from multipotent stem cells located near the base of crypt. The continual renewal of epithelial cells along CVA is accompanied by functional specialization and ensures the functions of small intestine [12]. The intestinal upper villus epithelial cells represent the differentiated epithelial cells and play key role in digesting and absorbing lumenal nutrients [12]. Fan et al. showed that the activities of alkaline phosphatase, aminopeptidase N, sucrase, lactase, and Na^+^/K^+^-ATPase in the small intestinal epithelial cells were increased from crypt to villi in piglets, which suggests that the upper villus epithelial cells play the key role in digesting nutrients [14]. Moreover, the mRNA expression of *EAAC1*, *SLC6A19*, and *SGLT1*, the transporters of L-glutamate, L-glutamine, and glucose, respectively, was increased during the epithelial cells differentiation along the CVA in the small intestine of piglets [15, 16]. These results also indicated that the epithelial cells in the upper villi paly important roles in absorbing nutrients in piglets. However, studies designed to test the effects of weaning on the small intestinal digesting and absorbing functions of piglets were used entire intestinal segments or mucosa. To our knowledge, there were no studies about the effects of weaning on upper villus epithelial cells in piglets. The proteomics has become a powerful tool for studying the expression of proteins in a global manner. The iTRAQ (isobaric tags for relative and absolute quantification) technique, as one of the powerful technologies of proteomics, is widely use for discovering physiology and function changes as it can be used to identify and relatively quantify the expression of proteins [17, 18]. In the present study, we hypothesized weaning will affect the physiology and functions of upper villus epithelial cells. Thus, the objective of the present study was to determine the physiology and functions of epithelial cells in the upper villi of piglets during post-weaning period usingiTRAQ technique.

## Materials and Methods

### Reagents

DL-β-Hydroxybutyrate sodium salt was purchased from J&K Chemical (Ltd., USA). Trypsin was procured from Promega (Madison, WI, USA). iTRAQ-reagent was purchased from Applied Biosystems (Foster City, CA, USA). Bovine serum alumin (BSA, fraction V), phenylmethylsulfonyl fluoride (PMSF), dithiothreitol (DTT), and other chemical were obtained from Sigma-Aldrich (St. Louis, MO, USA) unless otherwise stated.

### Animals and intestinal upper villus epithelial cells isolation

A total of 40 piglets (Duroc × [Landrace × Yorkshire]) from 8 litters (5 piglets per litter) were used in the present experiment. The piglets were weaned at the age of 14 d and fed creep diet that met the National Research Council nutrient specifications for 5 to 10 kg BW pigs as previously described[19, 20]. The piglets from the same litter were located in the same pen. Piglets had free access to feed and drinking water at all times throughout the experimental period. One piglet from each litter was maintained under general anesthesia and sacrificed by an intravenous (jugular vein) injection of 4% sodium pentobarbital solution (40 mg/kg body weight) at 0 d (w0d), 1 d (w1d), 3 d (w3d), 5 d (w5d), and 7 d (w7d) after weaning, respectively. The intestinal upper villus epithelial cells were isolated using the distended intestinal sac method as previously described with slight modifications [14, 20]. The divided mid-jejunum segments were rinsed thoroughly with ice-cold physiological saline solution and incubated at 37°C for 30 min with oxygenated phosphate buffered saline (PBS). The jejunum segments were then filled with oxygenated isolation buffer (5 mM Na2EDTA, 10 mM HEPES pH 7.4, 0.5 mM DTT, 0.25% BSA, 2.5 mM D-glucose, 2.5 mM L-glutamine, 0.5 mM dl-β-hydroxybutyrate sodium salt, oxygenated with an O2/CO2 mixture (19:1, v/v)) and incubated at 37°C for 40 min, and the isolation buffers were collected and centrifuged at 400 g for 4 min at 4°C. The collected cells were then washed twice with an oxygenated cell suspension buffer (10 mM HEPES, 1.5 mM CaCl2, 2.0 mM MgCl2, pH 7.4) and the cells were retained through centrifugation at 400 g for 4 min at 4°C. The isolated cells were immediately frozen in liquid nitrogen and then stored at -80°C until analysis. The collected cells were intestinal upper villus cells. The experimental design and procedures used in this study were carried out in accordance with the Chinese Guidelines for Animal Welfare and Experimental Protocols, and approved by the Animal Care and Use Committee of the Institute of Subtropical Agriculture at the Chinese Academy of Sciences.

### Sample preparation and isobaric labeling

The harvested cells were re-suspended and disrupted in lysis buffer composed of 7 M urea, 2 M thiourea, 4% w/v 3-[(3-Cholamidopropyl) dimethylammonio] propanesulfonate (CHAPS), 20 mMTributyl phosphate (TBP), and 0.2% Bio-lyte (pH 3–10), and a protease inhibitor cocktail (Roche Diagnostics Ltd, Mannheim, Germany). DNAse I and RNAse A were added to the lysate at final concentrations of 1 mg/mL and 0.25 mg/mL, respectively. After cell disruption, the protein solution was separated from the cell debris by centrifugation (12,000 × g, 5 min, 4°C). The crude protein extracts were further purified using the Ready Prep 2-D Cleanup Kit (Bio-Rad Laboratories, USA) and then underwent a reductive alkylation reaction. The protein concentration was determined using a 2-D Quant Kit (GE Healthcare, USA). Trypsin digestion and iTRAQ labeling were performed according to the manufacturer’s protocol (Applied Biosystems, Foster City, CA, USA). Briefly, 100 μg total protein of cell fraction was reduced and alkylated, digested overnight at 37°C with trypsin (Promega, Madison, WI, USA), and labeled with iTRAQ-reagents (Applied Biosystems, Foster City, CA, USA) as follows: W0d, iTRAQ reagent 115; W1d, iTRAQ reagent 116; W3d, iTRAQ reagent 117; W5d, iTRAQ reagent 118; W7d, iTRAQ reagent 121.

### Peptide fractionation and LC-MS/MS acquisition

The isotopically labeled samples were pooled and fractionated into 12 fractions by an Ultremex SCX column containing 5-μm particles (Phenomenex, USA). The eluted fractions were then desalted using a Strata X C18 column (Phenomenex, USA) and dried under vacuum. The final average peptide concentration in each fraction was about 0.25 μg/μL. Dried peptides were stored at -80°C before MS analysis. A nanospray ion source (Waters, USA) system coupled with Triple TOF was used for analytical separation. Microfluidic traps and nanofluidic columns packed with Symmetry C18 (5 μm, 180 μm × 20 mm) were utilized for online trapping, desalting, and nanofluidic columns packed with BEH130 C18 (1.7 μm, 100 μm × 100 mm) were employed in analytical separation. Solvents were composed of water/acetonitrile/formic acid (A: 98/2/0.1%; B: 2/98/0.1%). A portion of a 2.25 μg (9 μL) sample was loaded, and trapping and desalting. At a flow rate of 300 nL/min, analytical separation was established by maintaining 5% B for 1 min. In the following 64 min, a linear gradient to 35% B occurred in 40 min. Following the peptide elution window, the gradient was increased to 80% B in 5 min and maintained for 5 min. Initial chromatographic conditions were restored in 2 min.

Data acquisition was performed with a Triple TOF 5600 System (AB SCIEX, USA) fitted with a Nanospray III source (AB SCIEX, USA) and a pulled quartz tip as the emitter (New Objectives, USA). Data was acquired using an ion spray voltage of 2.5 kV, curtain gas of 30 Psi, nebulizer gas of 15 Psi, and an interface heater temperature of 150°C. The MS was operated with a RP ≥ 30,000 FWHM for the TOF MS scans. For information dependant acquisition (IDA), survey scans were acquired in 250 ms and as many as 30 product ion scans were collected if they exceeded a threshold of 120 counts per second (counts/s) with a 2+ to 5+ charge-state. The total cycle time was fixed to 3.3 s and the Q2 transmission window was 100 Da for 100%.

Four time bins were summed for each scan at a pulser frequency value of 11 kHz through monitoring of the 40 GHz multichannel TDC detector with four-anode/channel detection. A sweeping collision energy setting of 35 ± 5 eV coupled with iTRAQ adjust rolling collision energy was applied to all precursor ions for collision-induced dissociation. Dynamic exclusion was set for 1/2 of the peak width (18 s), and the precursor was then refreshed off the exclusion list.

### Database analysis and quantification

Mascot software (version 2.3.02, Matrix Science) was used to simultaneously identify and quantify proteins. Searches were made against the NCBI non-redundant database consisting of Susscrofa proteins. Spectra from the 12 fractions were combined into one MGF (Mascot generic format) file after the raw data was loaded, and the MGF file was searched. The search parameters were: i) trypsin was chosen as the enzyme with one missed cleavage allowed; ii) the fixed modifications of carbamidomethylation were set as Cys; iii) peptide tolerance was set as 0.05 Da, and MS/MS tolerance was set as 0.1 Da. An automatic decoy database search strategy was employed to estimate the false discovery rate (FDR). The FDR was calculated as the false positive matches divided by the total matches. In the final search results, the FDR was less than 1.5%. The search results were passed through additional filters before data exportation. For protein identification, the filters were set as follows: significance threshold *P* < 0.05 (with 95% confidence) and ion score or expected cutoff less than 0.05 (with 95% confidence). For protein quantitation, the filters were set as follows: “median” was chosen for the protein ratio type; the minimum precursor charge was set to 2+ and minimum peptides were set to 2; only 2 and > 2 unique peptides were used to quantify proteins. The median intensities were set as normalization, and outliers were removed automatically. The peptide threshold was set as above for identity. In present study, a protein with ≥ 1.2-fold or ≤ 0.8-fold difference between W1d, W3d, W5d or W7d and W0d and a P-value ≤ 0.05 was regarded as being differentially expressed.

### Bioinformatics analysis

Functional annotations of the differentially expressed proteins were conducted using Blast2GO program against non-redundant database consisting of Susscrofa proteins [21]. The KEGG database [22] and the WEGO program [23] were used to classify and group the differentially expressed proteins. The cluster of differentially expressed proteins was done by Cluster 3.0 using k-means clustering [24]. The up-regulated (Up) and down-regulated (Down) proteins enriched groups were selected. Cellular component, molecular function, and biological process ontology of the Up proteins and Down proteins were performed using WEGO program (S2 Text). The GO terms with a *P*-value ≤ 0.05 (Pearson Chi-Square test between the protein numbers of Up and Down proteins) were selected [23].

### RNA extraction and real-time quantitative PCR

Approximately 100 mg of cells from each piglet was ground to fine powder with a mortal and liquid nitrogen. The TRIZOL reagent (Invitrogen, Carlsbad, CA) was used to isolate total RNA from cells, and the isolated RNA was then treated with DNaseI (Invitrogen,Carlsbad, CA) according to the manufacturer's instructions. The RNA quality was checked by 1.2% agarose gel electrophoresis, after staining with ethidium bromide, and then cDNA was reverse transcribed and amplified by RT-PCR [25]. Oligo 6.0 software (Molecular Biology Insights, CO, USA) was used to design primers (Table A in S1 Text). The ABI 7900HT Fast Real-Time PCR System (Applied Biosystems, Carlsbad, CA) was used for real-time quantitative PCRanalyses. A total volume of 10 μL (5 μL SYBR Green mix, 1 μL 4 × diluted cDNA,0.2 μL50× ROX Reference Dye, 0.2 μL forwardprimer, 0.2 μL reverse primer, and 3.4 μLddH_2_O). After a pre-denaturation process (10 s at 95°C), an amplification process with 40 cycles, which was consisted of 95°C for 5 s, 60°C for 20 s, was performed. The amplificationprocess was followed by a melting curve program (60–99°C with a heating rate of 0.1°C/s and fluorescence collection). The amplification of *β-actin* was used to normalize the expression of the interestedgenes.The relative mRNA expression ratio (R) of the target gene was calculated by *R* = 2^−ΔΔ*Ct*(*sample*−*control*)^, where −ΔΔ*Ct*(*sample*−*control*) = (*Ct of* target ge*ne*−*Ct of GAPDH*)_*sample*_ − (*Ct of* target ge*ne*−*Ct of GAPDH*)_*control*_. The efficiency of real-time quantitative PCR was measuredby amplifying a dilution series (according to the equation 10^(−1/slope)^) of cDNA [26]. The mRNA of target genes and *GAPDH* was amplified with comparable efficiencies. In negative controls, the cDNA sample was replaced by water.

### Western blotting analysis

Total protein was extracted using ice-cold RIPA buffer (150 mM NaCl, 1% Triton X-100, 0.5% sodium deoxycholate, 0.1% SDS, 50 mM Tris-HCl at pH 7.4; Biyuntian, Shanghai, China), plus a protease inhibitor cocktail (Roche, Shanghai, China) and phosphatase inhibitors (Thermo Scientific, Bremen, Germany). After centrifugation at 10,000 × *g* and 4°C for 10 min, the protein concentration in the supernatant fluid was determined using a Bicinchoninic Acid assay (Beyotime Biotechnology, China). All samples were adjusted to an equal protein concentration and then diluted with 2 × loading buffer [0.63 ml of 0.5 M Tris-HCl (pH 6.8), 0.42 ml 75% glycerol, 0.125 g sodium dodecyl sulfate (SDS), 0.25 ml β-mercaptoethanol, 0.2 ml 0.05% solution of bromophenol blue, and 1 ml water] to a final volume of 2.5 ml and heated in boiling water for 5 min. Soluble proteins were subjected to SDS-PAGE, and transferred to PVDF membranes (Millipore, Billerica, MA), blocked with 5% nonfat milk in TBS-0.05% Tween-20 for 1 h and incubated overnight with primary antibodies followed by horseradish peroxidase-linked secondary antibodies (Santa Cruze). The bound antibodies were using enhanced chemiluminescence (Applygen Technologies Inc., Beijing, China) for detection [26]. The following antibodies were used for western blot analysis. Antibodies for eIF4E, I-FABP, Bcl2, and Caspase3 were purchased from Santa Cruz Biotechnology (Santa Cruz, CA), and antibodies for S6K, phospho-S6K (Thr389), mTOR, phospho-mTOR (Ser2448), 4EBP1, phospho-4EBP1 (Thr70) and β-actin were purchased from Cell Signaling Technology (Cedarlane, ON, Canada). The abundance of the target proteins was normalized by β-actin. The Quantity-One software (Bio-Rad) was used to quantify the bands of each protein per sample.

### Statistical analysis

Data of real-time Quantitative PCR and western blotting analysis were analyzed using one-wayANOVA (SAS 9.3, Cary, NC), followed by Duncan’s multiple comparison test. The probability values < 0.05 were taken to indicate statistical significance.

## Results

### Changes in protein expression in intestinal upper villus epithelial cells of weaning piglets

A biological replicate sample was included in the iTRAQ experiment in w0d, w1d, w3d, w5d, and w7d groups. A total of 898 differentially expressed proteins were identified in the present study. Cellular component GO enrichment analysis showed that the differentially expressed proteins were mainly involved in cell, cell part, organelle, organelle part, macromolecular complex, membrane-enclosed lumen, envelope, extracellular region, and extracellular region part (Fig A in S1 Text). Molecular function GO enrichment analysis showed that the differentially expressed proteins were mainly involved in binding, catalytic activity, structural molecule activity, transporter activity, enzyme regulator activity, electron carrier activity, transcription regulator activity, and molecular transducer activity (Fig A in S1 Text). Biological process GO enrichment analysis showed that the differentially expressed proteins were mainly involved in cellular process, metabolic process, biological regulation, pigmentation, localization, multicellular organismal process, cellular component organization, response to stimulus, establishment of localization, and developmental process (Fig A in S1 Text). KEGG pathway enrichment analysis showed the differentially expressed proteins were mainly involved in ribosome, spliceosome, protein processing in endoplasmic reticulum, carbon metabolism, oxidative phosphorylation, biosynthesis of amino acids, proteasome, peroxisome, fatty acid metabolism, and glycolysis/gluconeogenesis, and so on (Fig B in S1 Text).

**Fig 1:**
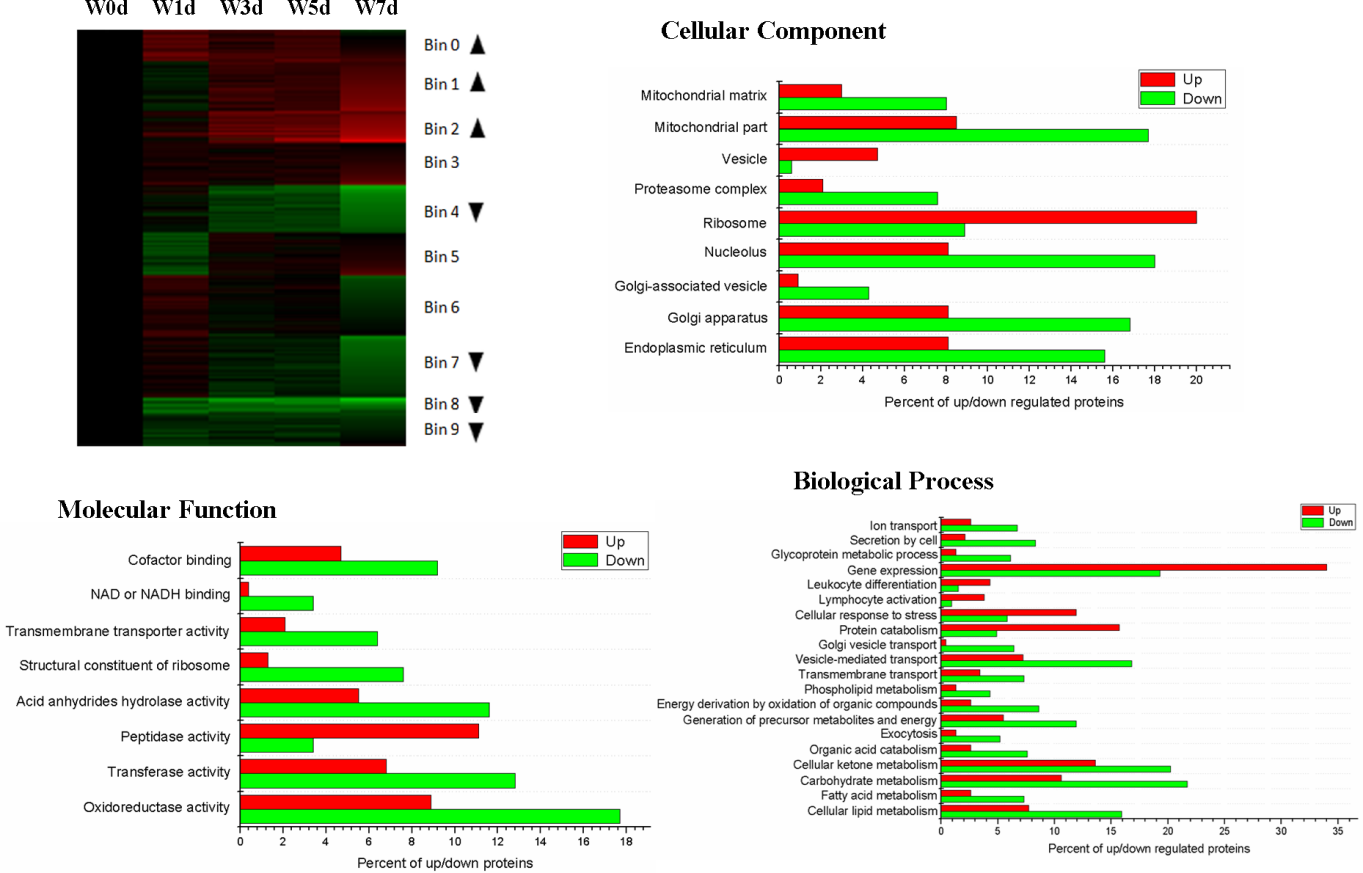
Functional categorization of up-regulated and down-regulated proteins in jejunal upper villus epithelial cells of weaning piglets. The differentially expressed proteins were clustered using Cluster 3.0 with k-means clustering. The up-regulated (Up) and down-regulated (Down) proteins enriched groups were selected (indicated by arrowheads). Cellular component, molecular function, and biological process ontology of the Up proteins (arrowheads pointing up) and Down proteins (arrowheads pointing down) were performed using WEGO program. The GO terms with a *P*-value ≤ 0.05 (Pearson Chi-Square test between the protein numbers of Up and Down proteins) were selected.

The differentially expressed proteins were clustered into 10 distinct groups (Bin 0 to Bin 9) using k-means clustering. Among these distinct groups, the expression of proteins in Bin0, Bin1 and Bin 3 were up-regulated after weaning, while the expression of proteins in Bin4, Bin 7, Bin8 and Bin 9 were down-regulated after weaning. The WEGO program was used to analyze GO enrichment of Up proteins and Down proteins, the GO terms that were significantly (*P*< 0.05) different between Up and Down groups were selected. On the ontology type of cellular component, the percent of proteins related to vesicle and ribosome in Up group was significantly greater than that in Down group, while the percent of proteins related to mitochondrial matrix, mitochondrial part, proteasome complex, nucleolus, golgi-associated vesicle, golgi apparatus, and endoplasmic reticulum in Up group was significantly less than that in Down group (Fig 1). On the ontology type of molecular function, the percent of proteins related to peptidase activity in Up group was significantly greater than that in Down group, while the percent of proteins related to cofactor binding, NAD or NADH binding, transmembrane transporter activity, structural constituent of ribosome, acid anhydrides hydrolase activity, transferase activity, and oxidoreductase activity in Up group was significantly less than that in Down group (Fig 1). On the ontology type of biological process, the percent of proteins related to gene expression, leukocyte differentiation, lymphocyte activation, cellular response to stress, and protein catabolism in Up group was significantly greater than that in Down group, while the percent of proteins related to ion transport, secretion by cell, glycoprotein metabolic process, golgi vesicle transport, vesicle-mediated transport, transmembrane transport, phospholipid metabolism, energy derivation by oxidation of organic compounds, generation of precursor metabolites and energy, exocytosis, organic acid metabolism, cellular ketone metabolism, carbohydrate metabolism, fatty acid metabolism, and cellular lipid metabolism in Up group was significantly less than that in Down group (Fig 1).

### Golgi vesicle transport, protein amino acid glycosylation, and secretion by cell

**Fig 2:**
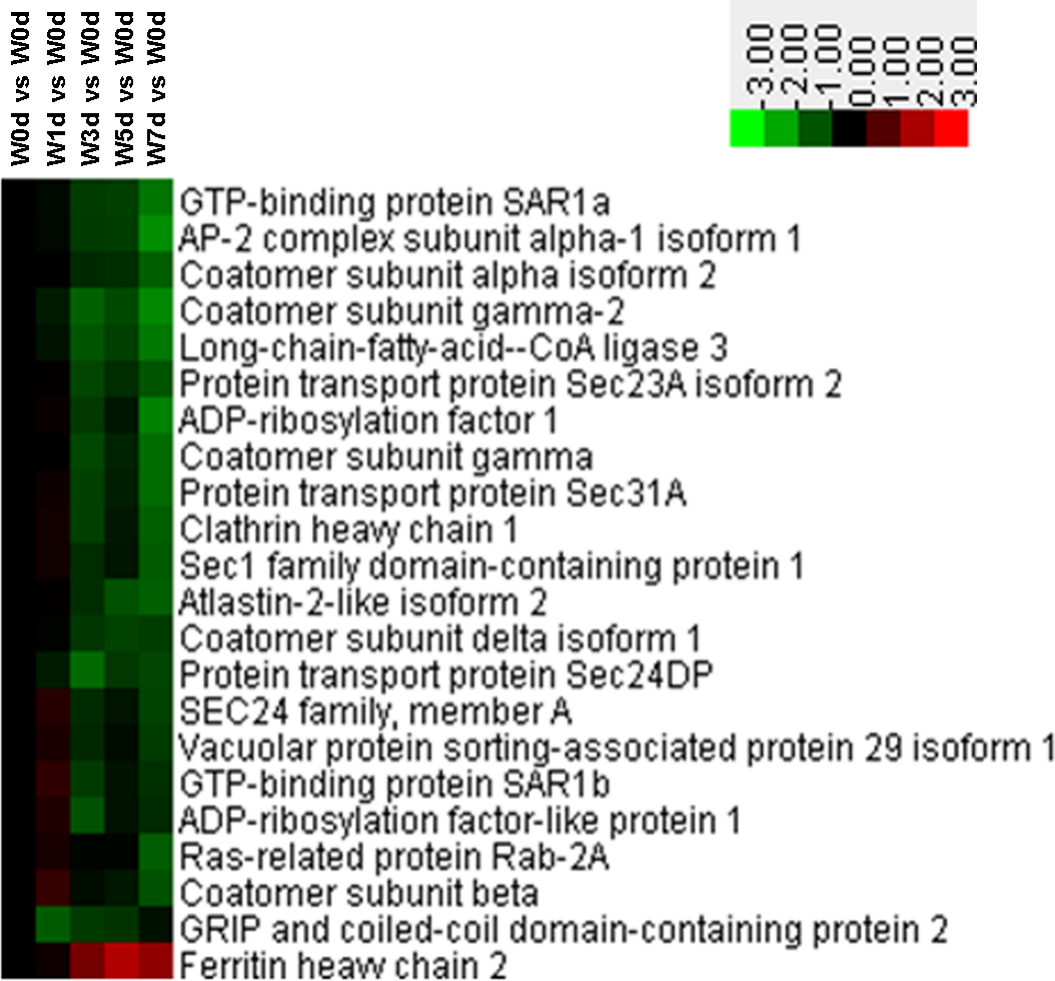
Proteins enriched in Golgi vesicle transport in jejunal upper villus epithelial cells of weaning piglets. There was a remarkable relationship (*P*< 0.05) in Golgi vesicle transport between Up and Down proteins based on WEGO program analysis. The Up and Down proteins enriched in Golgi vesicle transport were selected and clustered by Cluster 3.0.

**Fig 3:**
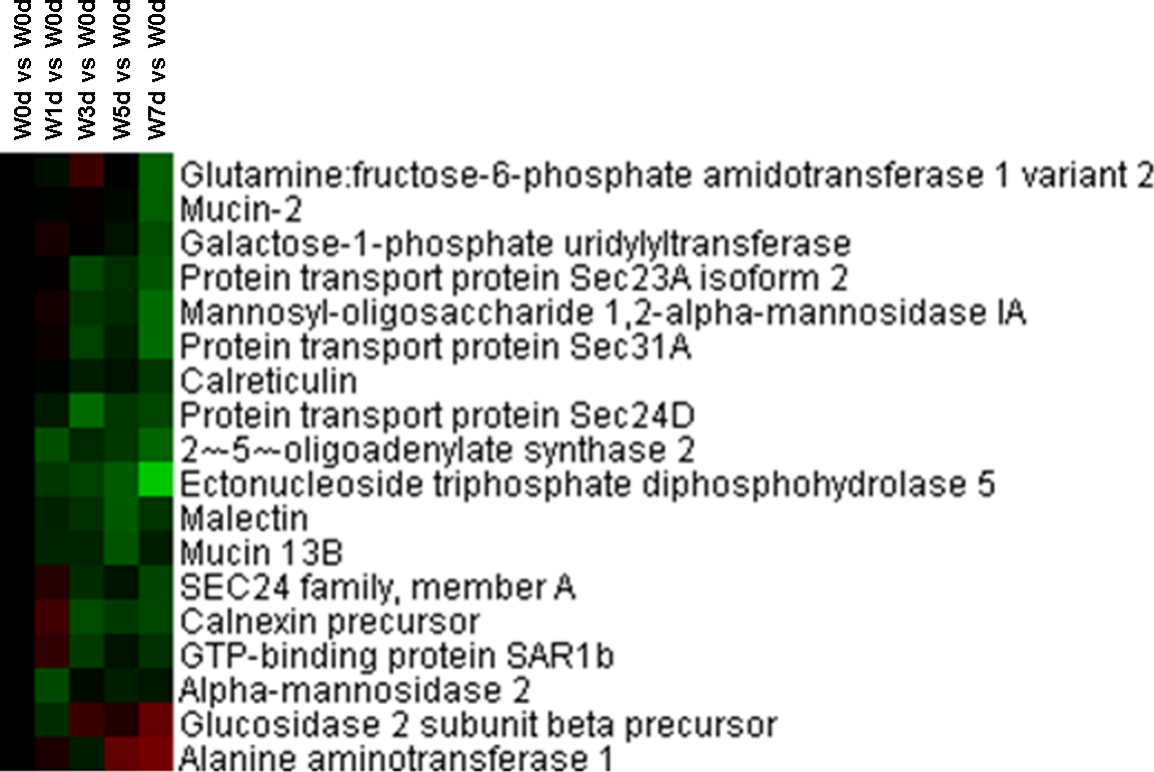
Proteins enriched in protein amino acid glycosylation in jejunal upper villus epithelial cells of weaning piglets. There was a remarkable relationship (*P*< 0.05) in protein amino acid glycosylation between Up and Down proteins based on WEGO program analysis. The Up and Down proteins enriched in protein amino acid glycosylation were selected and clustered by Cluster 3.0.

**Fig 4:**
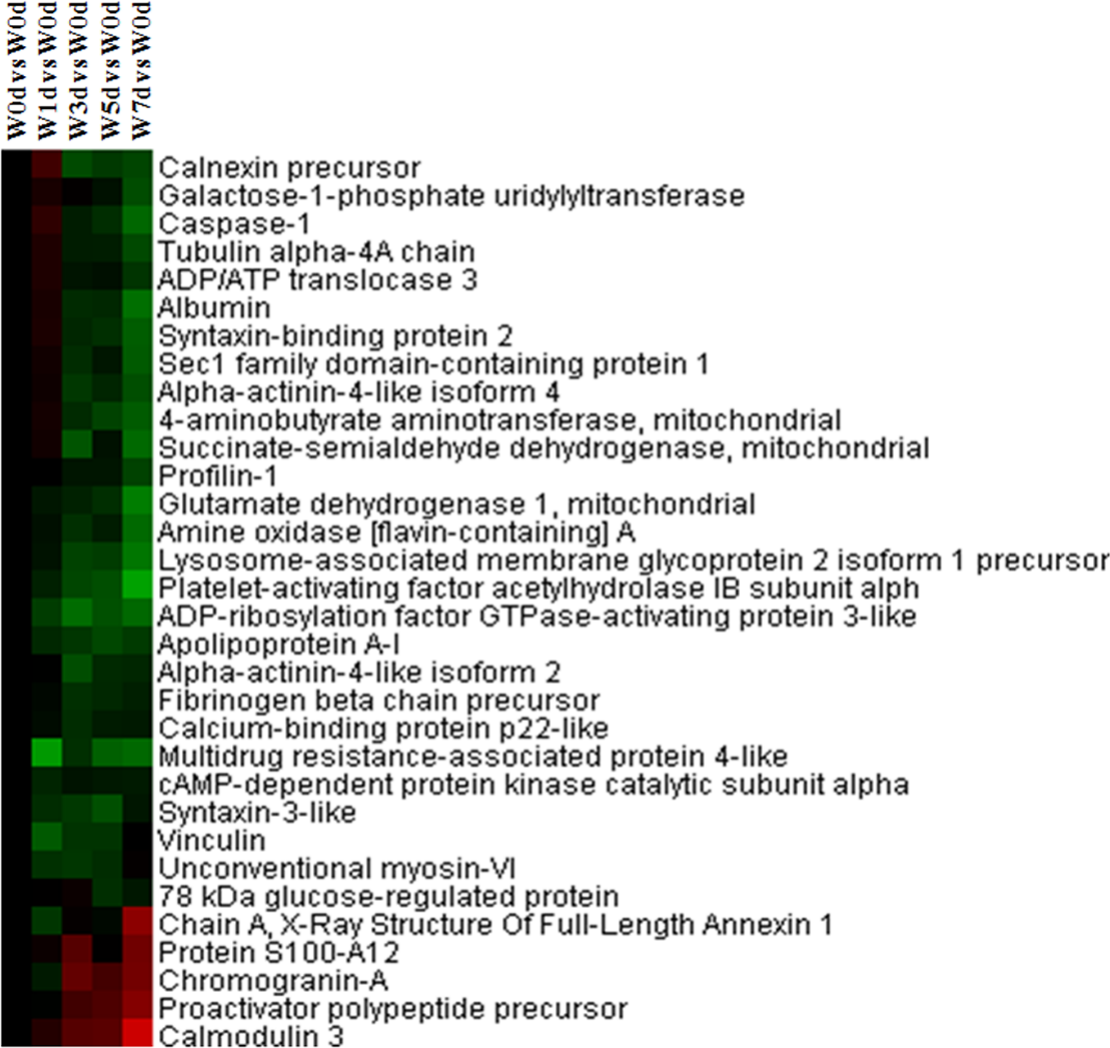
Proteins enriched in secretion by cell in jejunal upper villus epithelial cells of weaning piglets. There was a remarkable relationship (*P*< 0.05) in secretion by cell between Up and Down proteins based on WEGO program analysis. The Up and Down proteins enriched in secretion by cell were selected and clustered by Cluster 3.0.

Among the differentially expressed proteins in Up and Down groups, a total of 22 Golgi vesicle transport related proteins were identified. Of these proteins, the expression of 21 proteins was down-regulated after weaning, which mainly involved in retrograde vesicle-mediated transport (Golgi to ER), intra-Golgi vesicle-mediated transport, COPII coating of Golgi vesicle, and COPI coating of Golgi vesicle (Fig 2). A total of 18 protein amino acid glycosylation related proteins were identified from Up and Down groups, and the expression of 16 proteins (including mucin-2, mucin 13B, 2-5-oligoadenylate synthase 2, and glucosidase 2 subunit beta precursor and so on) was down-regulated after weaning (Fig 3). Thirty-two proteins with a role in secretion by cell were identified from the differentially expression proteins, with the majority of proteins down-regulated after weaning (Fig 4). These down-regulated proteins were mainly involved in platelet degranulation, protein secretion, and neurotransmitter secretion (Fig 4).

### Transmembrane transport, ion transport, and nucleotide catabolic process

**Fig 5:**
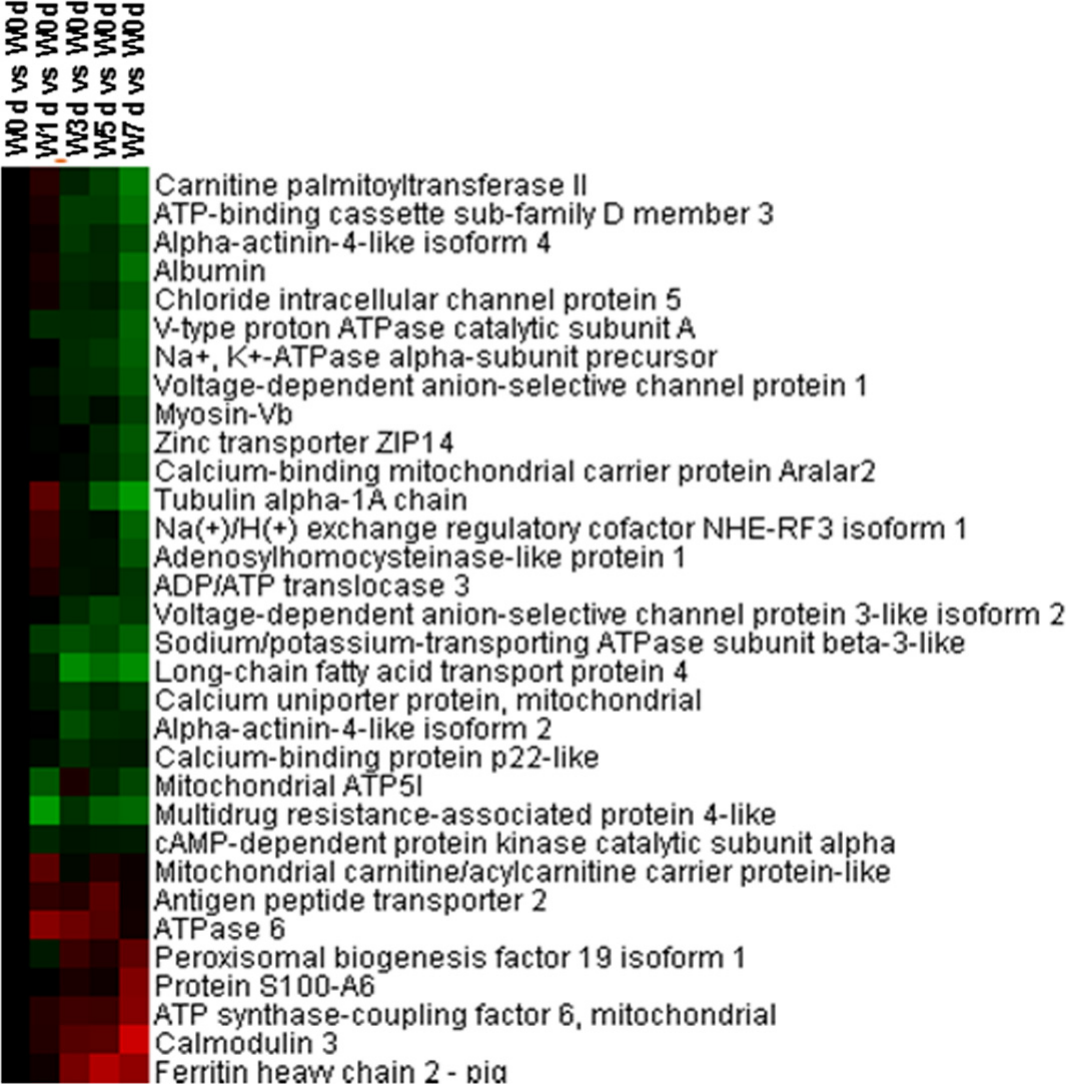
Proteins enriched in transmembrane transport in jejunal upper villus epithelial cells of weaning piglets. There was a remarkable relationship (*P*< 0.05) in transmembrane transport between Up and Down proteins based on WEGO program analysis. The Up and Down proteins enriched in transmembrane transport were selected and clustered by Cluster 3.0.

**Fig 6:**
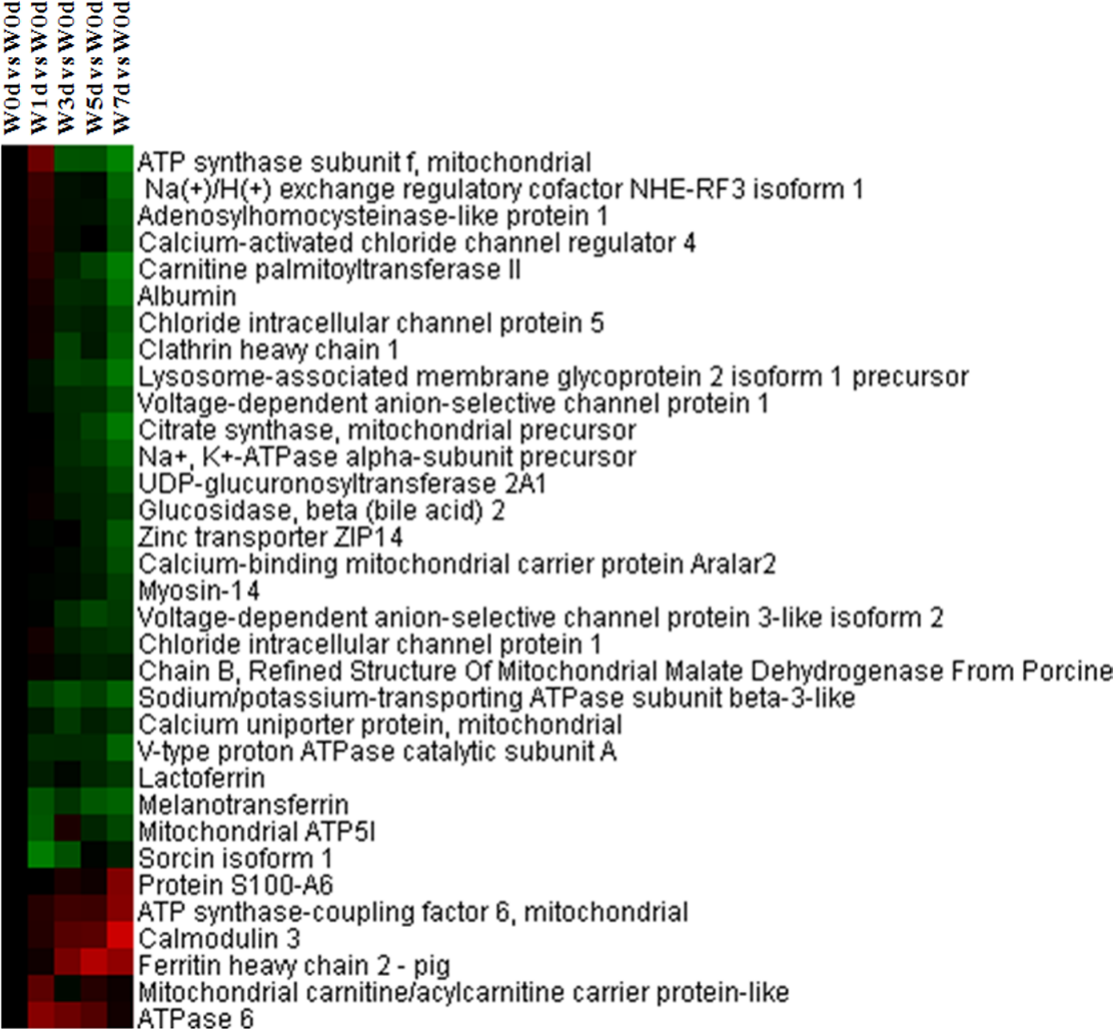
Proteins enriched in ion transport in jejunal upper villus epithelial cells of weaning piglets. There was a remarkable relationship (*P*< 0.05) in ion transport between Up and Down proteins based on WEGO program analysis. The Up and Down proteins enriched in ion transport were selected and clustered by Cluster 3.0.

**Fig 7:**
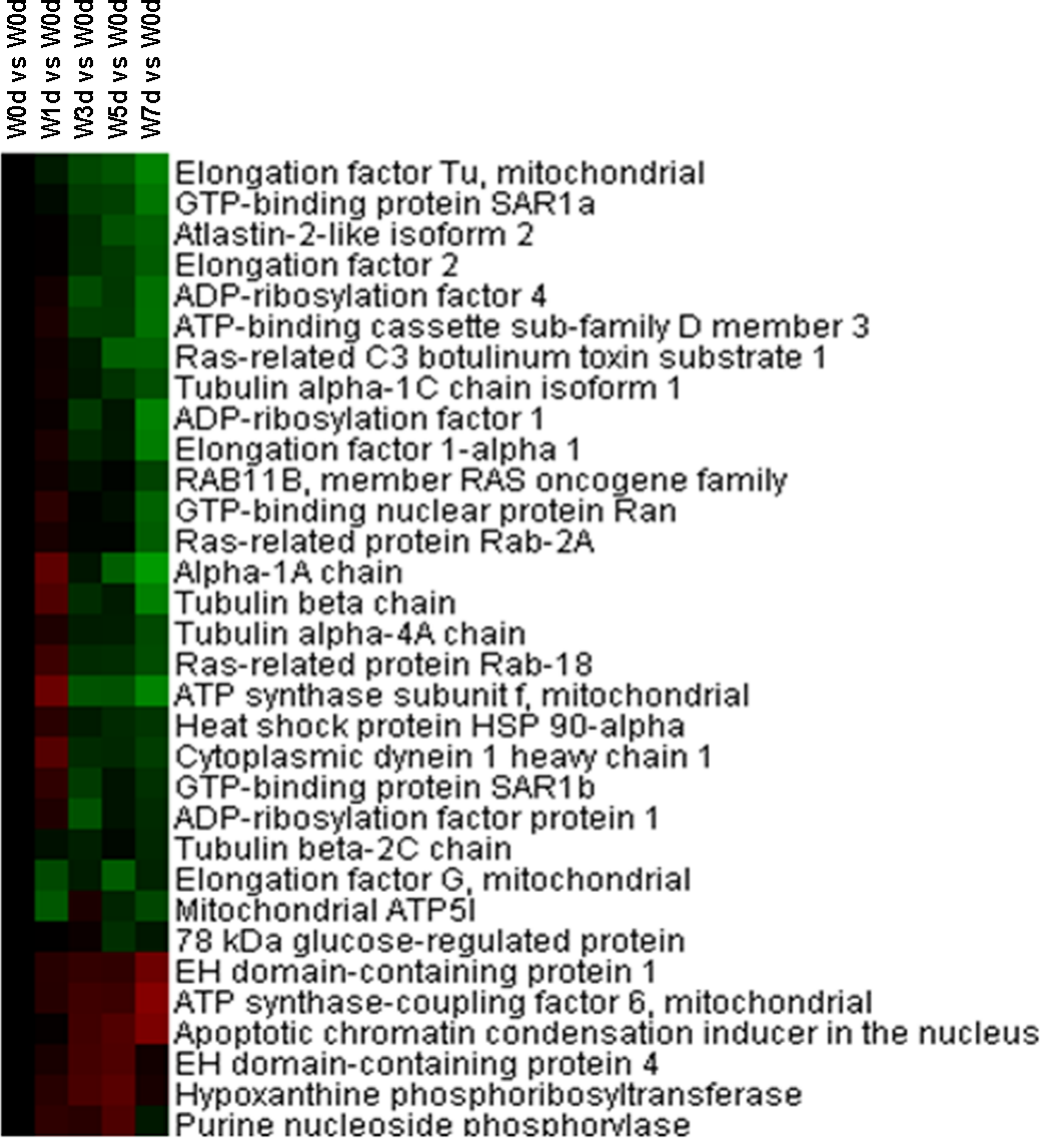
Proteins enriched in nucleotide catabolic process in jejunal upper villus epithelial cells of weaning piglets. There was a remarkable relationship (*P*< 0.05) in nucleotide catabolic process between Up and Down proteins based on WEGO program analysis. The Up and Down proteins enriched in nucleotide catabolic process were selected and clustered by Cluster 3.0.

A total of 32 proteins involved in transmembrane transport were identified from Up and Down groups, with 24 proteins down-regulated in expression after weaning, and the down-regulated proteins were mainly involved in amino acid transport and ion transmembrane transport (Fig 5). Thirty-three proteins with a role in ion transport were identified from Up and Down groups, and 27 proteins were down-regulated after weaning, which were mainly related to chloride transport, calcium ion transport, potassium ion transport, and metal ion transport (Fig 6). A total of 32 proteins related to nucleotide catabolic process were identified from Up and Down groups, and 26 proteins were down-regulated after weaning, with 20 proteins related to GTP catabolic process and 6 proteins related to ATP catabolic process (Fig 7).

### Translational initiation, mTOR signaling pathway, and differentiation and apoptosis

**Fig 8:**
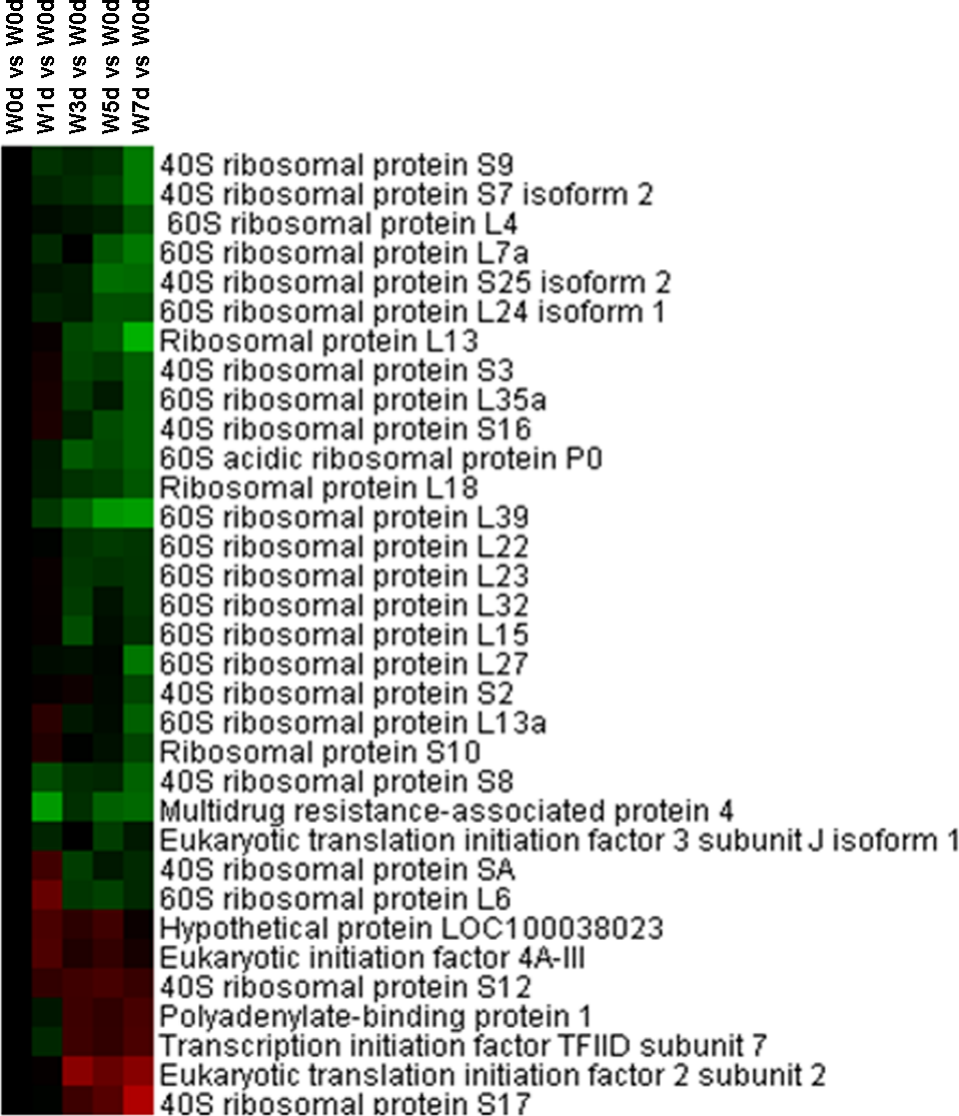
Proteins enriched in translational initiation in jejunal upper villus epithelial cells of weaning piglets. There was a remarkable relationship (*P*< 0.05) in translational initiation between Up and Down proteins based on WEGO program analysis. The Up and Down proteins enriched in translational initiation were selected and clustered by Cluster 3.0.

Thirty-three proteins related to translational initiation were identified from Up and Down groups, with 26 proteins down-regulated in expression after weaning. These included numerous components of 60S ribosomal protein and 40S ribosomal protein, and translation initiation factor (Fig 8). The abundance of proteins in mTOR signaling pathway, including 4E-BP1, p-4E-BP1, mTOR, p-mTOR, S6k, p-S6k, and eIF4E, was measured by Western Blotting. The results showed that the abundance of p-4E-BP1, S6k(1), p-S6k(1) and p-S6k (2) was reduced (*P*<0.05) afterweaning (Table 1, Fig C in S1 Text). Moreover, the expression of I-FABP was reduced (*P*<0.05) after weaning, and the abundance of bcl-2, caspase-3(1), caspase-3(2), and caspase-3(3)was also decreased (*P*<0.05) after weaning (Table 2, Fig D in S1 Text).

**Table 1:** Effects of weaning on the expression of proteins in mammalian target of rapamycin signaling pathway (mTOR) in jejunal upper villus epithelial cells of piglets.

Item	W0d	W2d	W3d	W5d	W7d	SEM	*P*-value
4E-BP1	0.652	0.715	0.797	0.696	0.685	0.019	0.418
p-4E-BP1	0.551	0.508	0.437	0.422	0.395	0.012	0.018
mTOR	0.280	0.295	0.298	0.328	0.226	0.009	0.078
p-mTOR	0.247	0.259	0.224	0.264	0.235	0.009	0.813
S6K(1)	0.280	0.246	0.229	0.217	0.209	0.007	0.095
S6K(2)	0.335	0.311	0.277	0.277	0.237	0.008	0.050
p-S6K(1)	0.189	0.169	0.123	0.121	0.067	0.005	0.001
p-S6K(2)	0.306	0.272	0.247	0.180	0.149	0.008	0.001
eIF4E	0.483	0.480	0.421	0.368	0.369	0.013	0.081

Values represent 6 pigs per treatment.

**Table 2:** Effect of weaning on the expression of I-FABP, Bcl-2, and caspase-3 in jejunal upper villus epithelial cells of piglets.

Item	W0d	W2d	W3d	W5d	W7d	SEM	*P*-value
I-FABP	0.375	0.408	0.313	0.285	0.279	0.009	0.004
Bcl2	0.407	0.350	0.246	0.235	0.218	0.010	0.001
Caspase3(1)	0.637	0.589	0.462	0.442	0.442	0.016	0.008
Caspase3(2)	0.761	0.654	0.486	0.425	0.437	0.016	0.001
Caspase3(3)	0.669	0.657	0.544	0.460	0.504	0.017	0.011

Values represent 6 pigs per treatment.

### Energy metabolism

**Fig 9:**
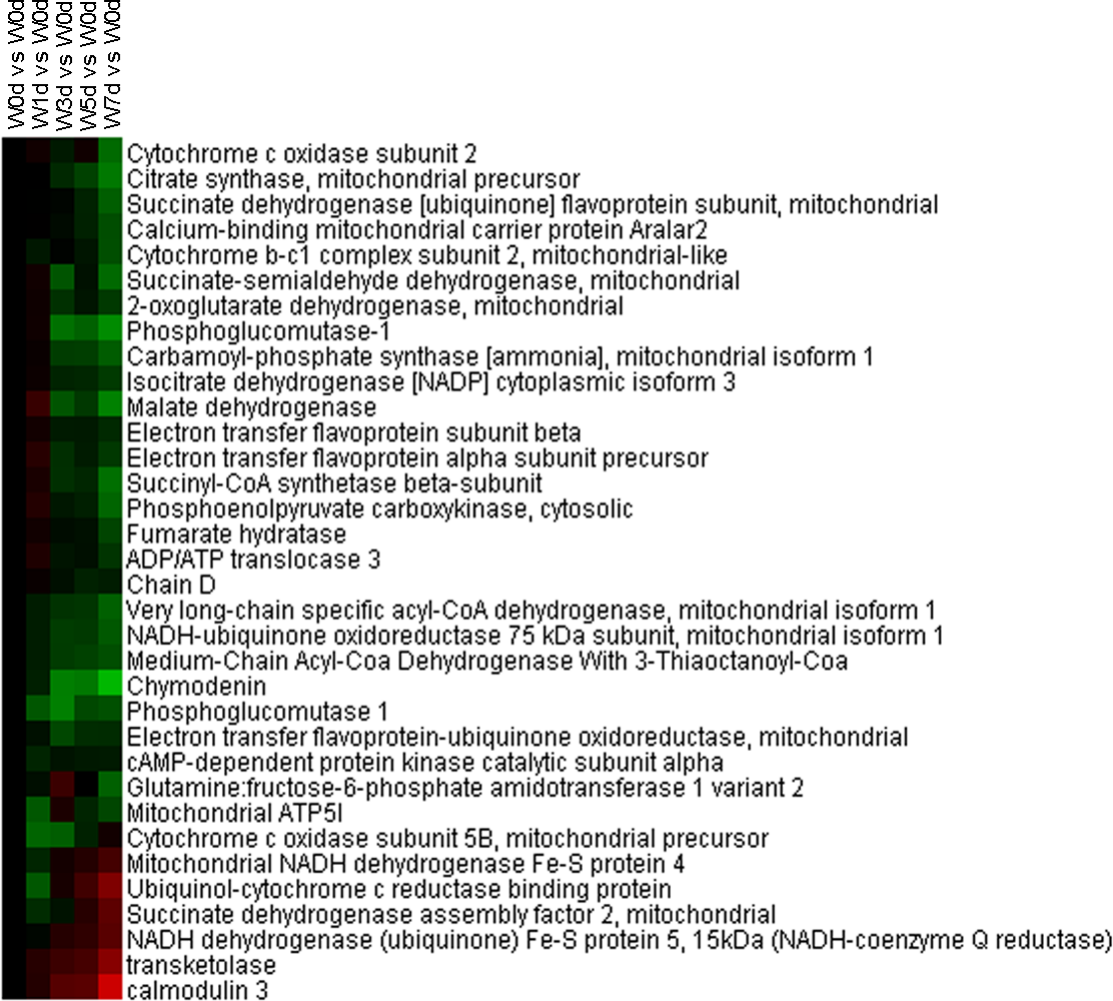
Proteins enriched in energy derivation by oxidation of organic compounds in jejunal upper villus epithelial cells of weaning piglets. There was a remarkable relationship (*P*< 0.05) in energy derivation by oxidation of organic compounds between Up and Down proteins based on WEGO program analysis. The Up and Down proteins enriched in energy derivation by oxidation of organic compounds were selected and clustered by Cluster 3.0.

**Fig 10:**
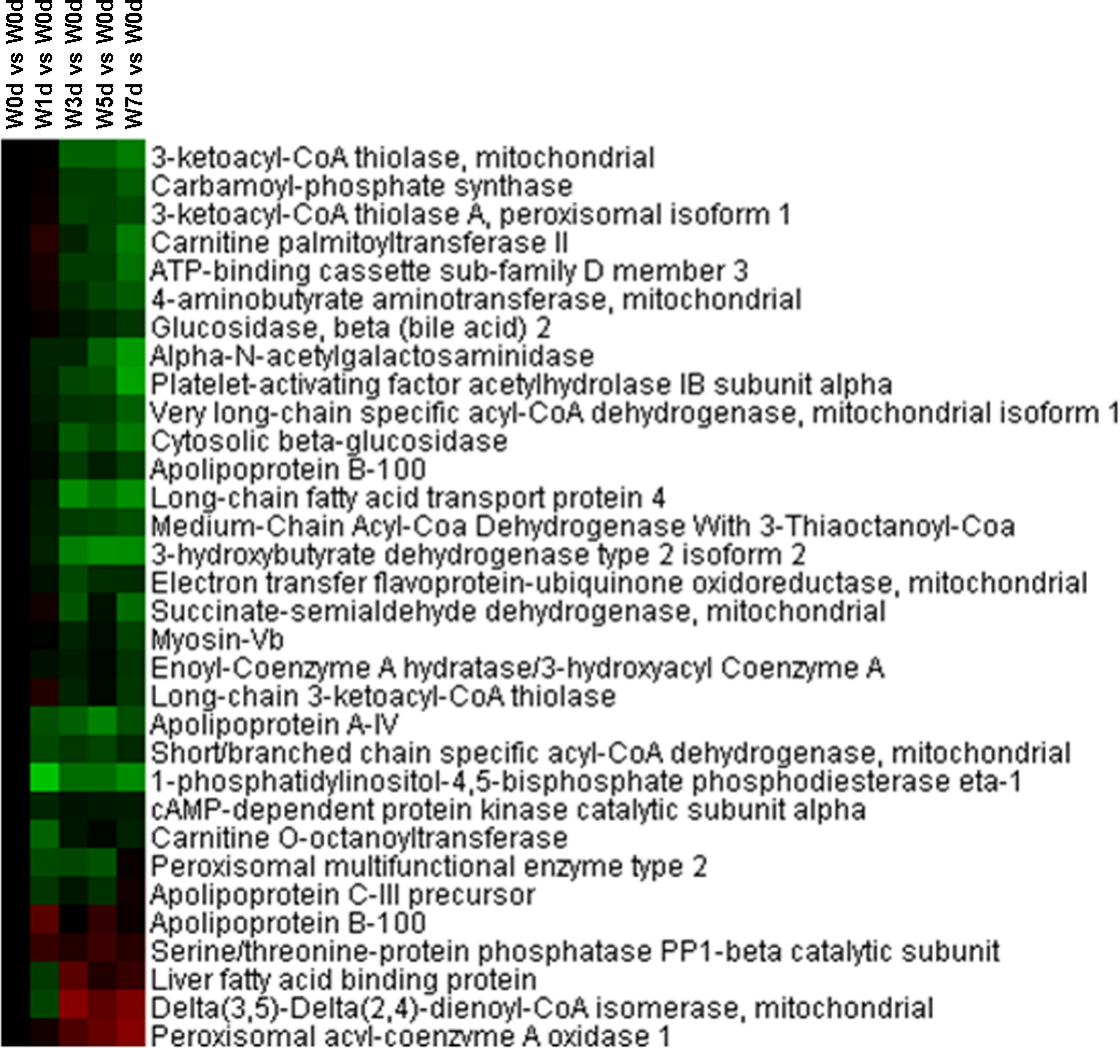
Proteins enriched in lipid catabolic process in jejunal upper villus epithelial cells of weaning piglets. There was a remarkable relationship (*P*< 0.05) in lipid catabolic process between Up and Down proteins based on WEGO program analysis. The Up and Down proteins enriched in lipid catabolic process were selected and clustered by Cluster 3.0.

**Fig 11:**
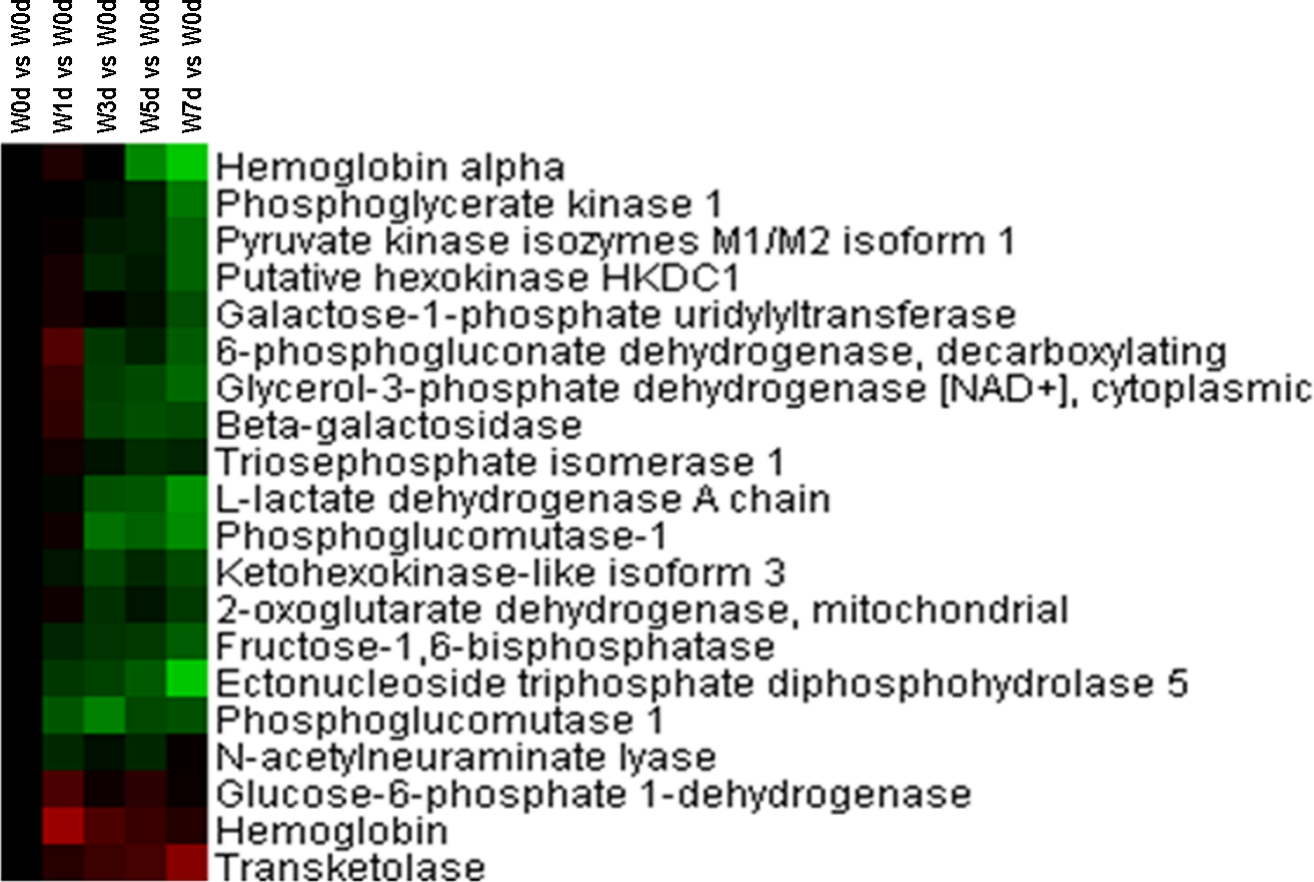
Proteins enriched in monosaccharide catabolic process in jejunal upper villus epithelial cells of weaning piglets. There was a remarkable relationship (*P*< 0.05) in monosaccharide catabolic process between Up and Down proteins based on WEGO program analysis. The Up and Down proteins enriched in monosaccharide catabolic process were selected and clustered by Cluster 3.0.

**Fig 12:**
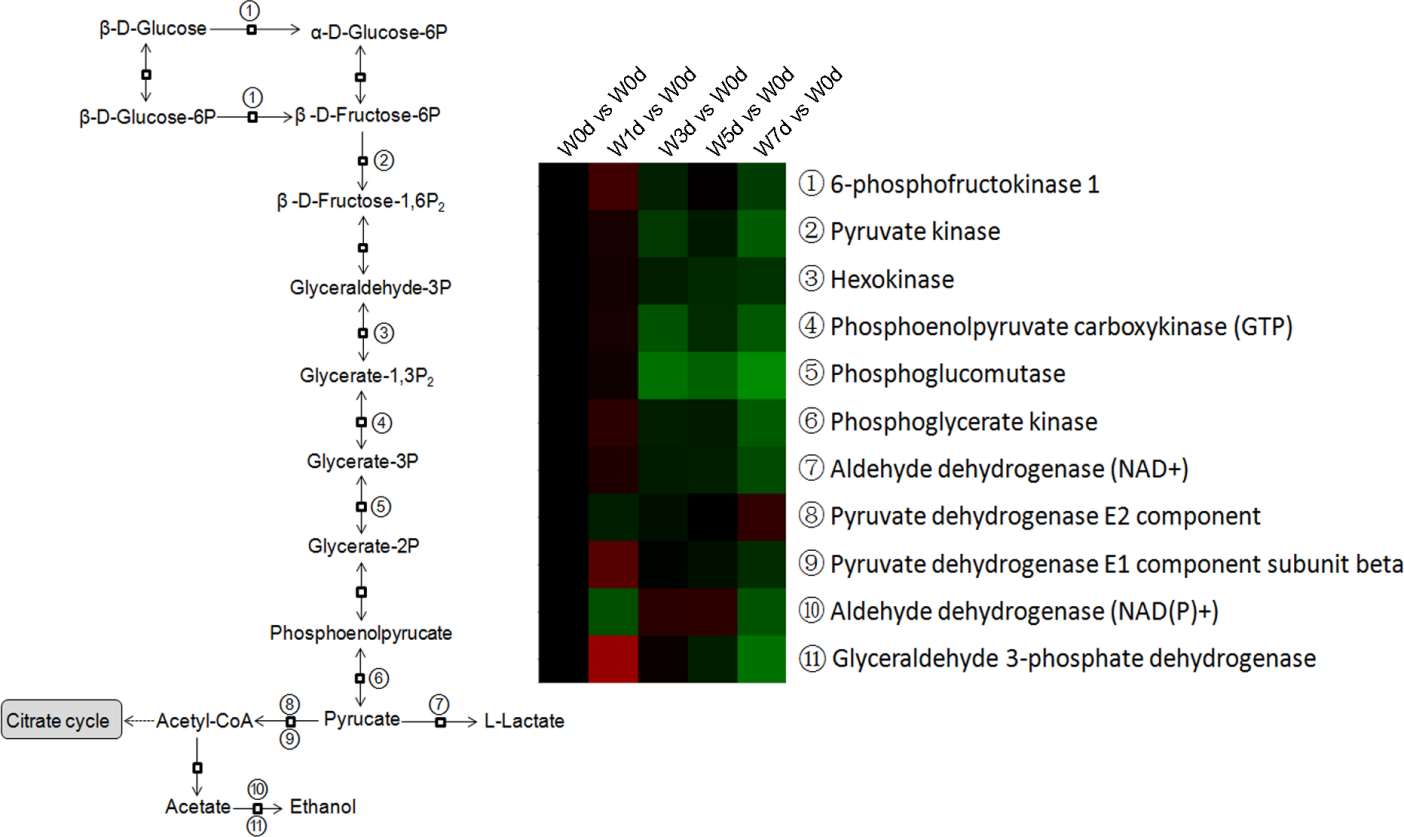
The enrichment of differentially expressed proteins in glycolysis pathway in jejunal upper villus epithelial cells of weaning piglets. A protein with ≥ 1.2-fold or ≤ 0.8-fold difference between W1d, W3d, W5d or W7d and W0d and a *P*-value ≤ 0.05 was regarded as being differentially expressed. The glycolysis pathway enrichment was performed using KEGG database.

**Fig 13:**
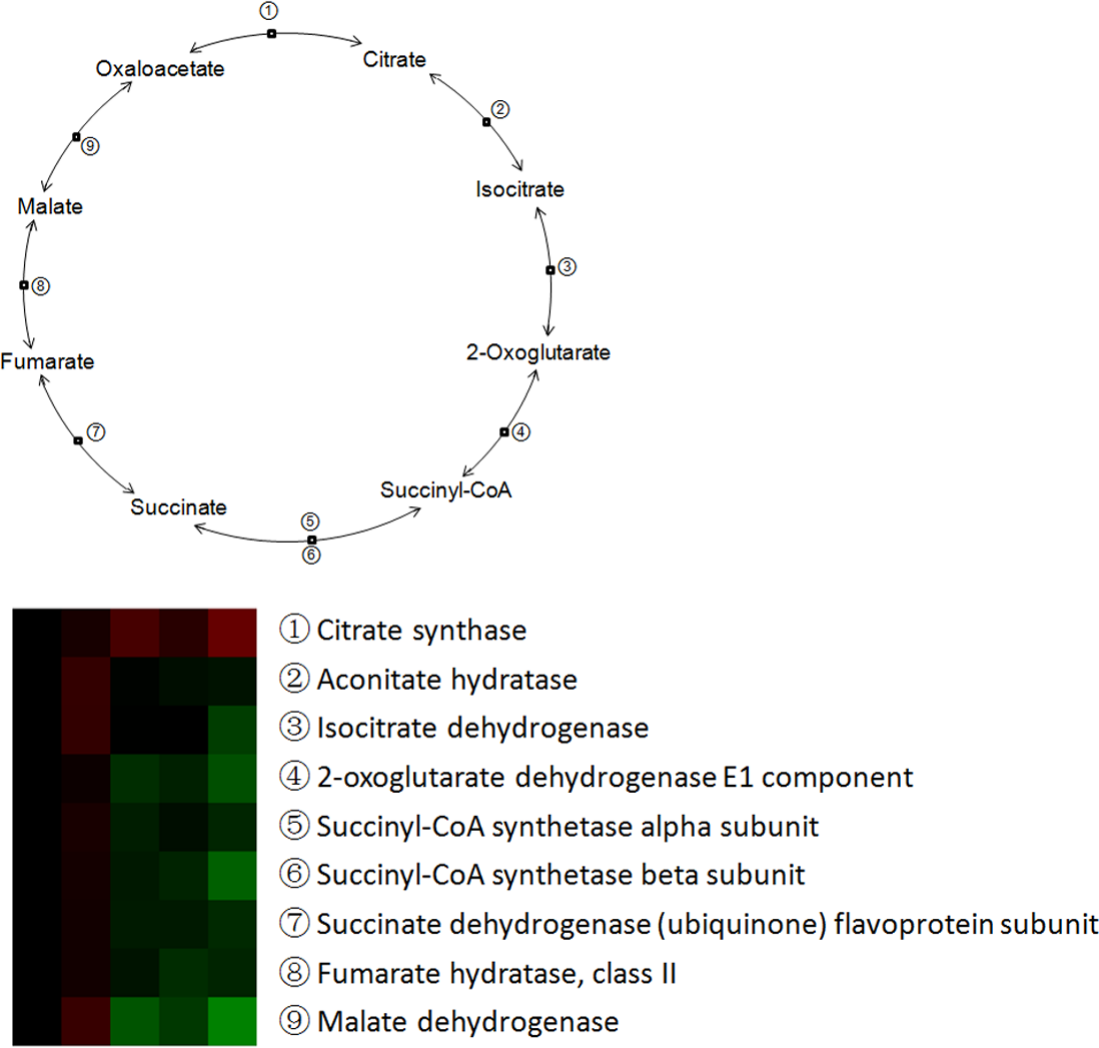
The enrichment of differentially expressed proteins in citrate cycle pathway in jejunal upper villus epithelial cells of weaning piglets. A protein with ≥ 1.2-fold or ≤ 0.8-fold difference between W1d, W3d, W5d or W7d and W0d and a *P*-value ≤ 0.05 was regarded as being differentially expressed. The citrate cycle pathway enrichment was performed using KEGG database.

A total of 34 proteins related energy derivation by oxidation of organic compounds were identified from Up and Down groups, and 28 proteins were down-regulated after weaning, which were mainly involved in respiratory electron transport chain, citrate acid cycle, and glycogen metabolic process (Fig 9). Thirty-two proteins with a role in lipid catabolic process were identified from Up and Down groups, with 27 proteins down-regulated in expression after weaning. The down-regulated proteins were mainly involved in fatty acid beta-oxidation and triglyceride catabolic process (Fig 10). Moreover, a total of 20 proteins involved in monosaccharide catabolic process were identified from Up and Down groups, and the expression of 17 proteins was reduced after weaning. These down-regulated proteins were mainly involved in glycolysis and galactose catabolic process (Fig 11). Proteins related to glycolysis were enriched using KEGG database, which showed that the expression of proteins in glycolysis was mainly reduced after weaning (Fig 12). In addition, most of identified proteins in citrate cycle were down-regulated after weaning excepted citrate synthase (Fig 13). The mRNA expression of genes related to glycolysis, fatty acids catabolism, and citrate cycle was measured by RT-PCR. The results showed that the mRNA expression of *PYK* (glycolysis), *CPT2*, *L-ACD*, and *ACO* (fatty acids catabolism), and *ICDH* and *OxoGDH* (citrate cycle) was elevated (*P*<0.05) after weaning (Table 3).

**Table 3:** The mRNA expression of genes related to glycolysis, fatty acids catabolism, and citrate cycle in jejunal upper villus epithelial cells of weaning piglets.

Item	W0d	W2d	W3d	W5d	W7d	SEM	*P*-value
*PYK*	1.031	1.048	1.146	1.342	1.498	0.043	0.041
*CISN*	1.025	1.025	1.032	1.121	1.067	0.033	0.941
*ICDH*	1.044	1.205	1.267	1.403	1.715	0.042	0.004
*OxoGDH*	1.030	1.149	1.233	1.481	1.419	0.039	0.035
*CPT1*	1.021	1.023	1.064	1.138	1.126	0.030	0.788
*CPT2*	1.039	1.266	1.291	1.384	1.437	0.033	0.047
*L-ACD*	1.033	1.242	1.277	1.500	1.530	0.040	0.023
*ACO*	1.024	1.239	1.396	1.545	1.462	0.034	0.004

*PYK*, Pyruvate kinase; *CISN*, Citrate synthase; *ICDH*, *i*socitrate dehydrogenase; *OxoGDH*, Oxoglutarate dehydrogenase;*CPT1*, Carnitine palmitoyltransferase 1; *CPT2*, Carnitine palmitoyltransferase 2; *L-ACD*, Long-chain acyl-CoA dehydrogenase; *ACO*,acyl-CoA oxidase.

Values represent 6 pigs per treatment.

## Discussion

Weaning was usually associated with gastrointestinal disorders and resulted in gastrointestinal dysfunction in post-weaning piglets [1, 27]. Previous studies were mainly conducted to test the effects of weaning on the structure and functions of intestine of weaning piglets, such as villus height, crypt depth, mucosal permeability, digestive enzymes activities, cytokine expression, and microflora [1, 8, 27–29]. Very few experiments were conducted to study the effects of weaning on the physiology of intestinal epithelial cells, especially the cells with different differentiation levels. Proteomics has been used to detect global proteins expression during various treatments and is particularly useful for screening the proteins involved in specific biological processes of interest, such as diseases, dietary treatments, and environmental changes [30]. In the present study, we isolated the differentiated epithelial cells from the jejunum of weaning piglets, and detected protein expression profiling of differentiated epithelial cells using proteomics. A total of 898 differentially expressed proteins were successfully identified from jejunal upper villus epithelial cells of 0d, 1d, 3d, 5d, and 7d post-weaning piglets. These proteins were mainly involved in cellular process, metabolic process, biological regulation, pigmentation, localization, multicellular organismal process, and so on. Zhu et al. compared gene expression profiling in the jejunum of weaning and age-matched suckling piglets and showed that the differentially expressed genes were mainly involved in cellular processes, biological regulation, metabolic processes, the regulation of biological processes, and so on [31]. These results indicated that weaning has similar effects on jejunal differentiated epithelial cells and entire jejunal segment. Hansson et al. showed that the differentially expressed proteins were enriched in carbohydrate metabolism, citrate cycle, fatty acid metabolism, electron transport, and amino acid metabolism, and so on during weaning period in the intestinal epithelial cells of mice [32]. From the results of the present experiment, the differentially expressed proteins were also enriched in carbohydrate metabolism, citrate cycle, fatty acid metabolism, and amino acid metabolism in jejunal differentiated epithelial cells of weaning piglets, while on enrichment in electron transport was also observed. The differences between the two studies may be resulted from the differences in animals (mice vs piglets) or epithelial cells (whole epithelial cells vs differentiated epithelial cells).

The weaning piglets usually have a dramatic reduction in nutrients (energy) intake leading to undernutrition. Although about 50% of piglets take the first post-weaning meal within 24 h, about 10% of piglets did not intake diet until 48 h [33]. The decrease in energy intake resulted in intestinal morphology alteration with reduction in villus height and increase in crypt depth [3, 34]. Moreover, the small intestine has a high rate of energy expenditure because the intake of nutrients by epithelial cells requires energy [35]. It was reported that the portal-drained viscera (including the intestine, pancreas, spleen, and stomach) contribute less than 5% of body weight, but they use more than 10% of whole-body energy expenditure [35, 36]. The results of the present experiment showed that the expression of proteins involved in energy metabolism (energy derivation by oxidation of organic compounds, lipid catabolic process, monosaccharide catabolic process, and citrate cycle) was mainly reduced in jejunal differentiated epithelial cells in post-weaning piglets. In addition, proteins involved in ATP and GTP catabolism were mainly down-regulated in piglets during the post-weaning period [37]. The expression of genes involved in lipid and glucose metabolism was increased in villus epithelial cells compared with crypt epithelial cells, and the energy of villus epithelial cells mainly came from lumenal nutrients [38, 39]. Therefore, the decrease in the expression of energy metabolism related proteins in jejunal differentiated epithelial cells of piglets may result from the decrease in energy intake during the post-weaning period, and energy supplementation may be an useful way to improve gastrointestinal functions of weaning piglets.

Although the protein expression of genes related to glucose catabolism, lipid catabolism, and citrate cycle was mainly decreased in post-weaning piglets, the mRNA expression of genes related to these cellular processes was mainly increased. The differences in protein and mRNA expression profiling of genes in glucose catabolism, lipid catabolism, and citrate cycle may be because of the expression of these genes was regulated at translational level as proteins related to translational initiation were mainly down-regulated in weaning piglets. Moreover, the abundance of p-4E-BP1, p-S6k, and eIF4E was decreased in piglets during post-weaning period. The 4E-BP1 directly regulates the rate of translation via binding to the cap-binding protein eIF4E and affecting the assembly of the translation initiation complex [40, 41]. When 4E-BP1 is phosphorylated, the binding between 4E-BP1 and eIF4E will be disrupted and the p-4E-BP1 will be released from the cap structure, then the translation process is initiated [40, 42]. The S6k is also involved in translational control of 5' oligopyrimidine tract mRNAs by phophorylating the S6 protein of the 40S ribosomal subunit [43]. Thus, weaning may through affecting the translation of proteins involved in glucose catabolism, lipid catabolism, and citrate cycle, and so on to affect the energy metabolism of jejunal differentiated epithelial cells. The results were consistent with a previous study which showed that the abundance of proteins related to fatty acid and glucose catabolism, citrate cycle, and mTOR signaling pathway was mainly decreased in jejunal differentiated epithelial cells of weaned piglets compared with those in suckling piglets [20].

The gastrointestinal epithelium is covered by a mucus gel composed predominantly of mucin glycoproteins [44]. The mucus is the anatomical site that the host first encounters gastrointestinal bacteria, and function to protect epithelial cells from infection, dehydration, and chemical or physical injury [44, 45]. The mucin glycoproteins were mainly synthesized and secreted by goblet cells in gut, and weaning was reported to result in lower levels of goblet cell densities and mucin in the villi [3, 11, 46]. Consistent with the previous studies, the results of the present experiment showed that proteins involved in protein amino acid glycosylation, including mucin-2, mucin 13B, and glucosidase 2 subunit beta precursor, and so on, were down-regulated in weaning piglets, which suggested that weaning destructed the protective coat on the surface of intestine. This may be one of the reasons of post-weaning diarrhoea in piglets. There are two types of mucins, membrane-bound and secreted, and the secretory mucins are secreted from the apical by baseline secretion or exocytosis [47]. Both types of mucins were synthesized or glycosylated in ER and Golgi apparatus [48]. The expression of proteins involved in Golgi vesicle transport and secretion by cell was reduced in the jejunal differentiated epithelial cells of post-weaning piglets. These results indicated that weaning may via affecting the function of Golgi and cell secretion to disrupt the mucus gel of intestine.

In weaned piglets, a decreased expression of genes related to cell proliferation and an increased expression of genes involved in apoptosis in jejunum were observed [31, 49]. It was proposed that weaning stress enhanced the apoptosis of epithelial cells, which was responsible for intestinal mucosal injury during post-weaning period [31, 50]. However, the results of the present experiment showed that the expression of capase-3 and bcl-2, two apoptosis-related marker proteins, was reduced in jejunal differentiated epithelial cells of post-weaning piglets. These results implied that the apoptosis of upper villus epithelial cells was reduced in piglets during post-weaning period. The differences in the expression profiling of genes related apoptosis between previous and the present experiments may resulted from the differences in tissue samples as the previous experiments used whole jejunal segments while the present experiment used jejunal differentiated epithelial cells [31, 49]. Moreover, the expression of I-FABP, a differentiating marker of intestinal epithelial cells [51], was also decreased in weaning piglets, which suggested that the differentiation levels of villus upper epithelial cells were decreased in piglets during the post-weaning period. In addition, the expression of proteins related to transmembrane transport and ion transport was mainly reduced in weaning piglets. The dysfunction of digestive and absorptive abilities of weaning piglets may partly resulted from the relatively undifferentiated epithelial cells in jejunum as the differentiated epithelial cells play key roles in digesting and absorbing lumenal nutrients [12]. Further studies are needed to test the mechanism of weaning on the differentiation of intestinal epithelial cells.

In conclusion, the results of the present study showed that the expression of proteins related to various cellular metabolic or biological processes, such as energy metabolism, Golgi vesicle transport, protein amino acid glycosylation, secretion by cell, transmembrane transport, ion transport, nucleotide catabolic process, translational initiation, mTOR signaling pathway, and differentiation and apoptosis, and so on, was mainly down-regulated in jejunal differentiated epithelial cells of piglets during the post-weaning period. These results indicate weaning has very complicated effects on the physiology of jejunal differentiated epithelial cells in piglets, and provide potential new directions for exploring the mechanism of weaning stress on the functions of intestine and means to improve the functions of intestine in weaning piglets.

## Supporting Information

S1 Text Supplementary tables and figures. **Table A.** Primers used for PCR analysis. **Fig A** Cellular component, molecular function, and biological process ontology of differentially expressed proteins in jejunal upper villus epithelial cells of piglets during the post-weaning period. **Fig B** KEGG pathways enrichment of differentially expressed proteins in jejunal upper villus epithelial cells of weaning piglets. **Fig C** Effects of weaning on the expression of proteins in mammalian target of rapamycin signaling pathway (mTOR) in jejunal upper villus epithelial cells of piglets. The expression of proteins in mTOR signaling pathway was measured using Western blotting. **Fig D** Effect of weaning on the expression of I-FABP, Bcl-2, and caspase-3 in jejunal upper villus epithelial cells of piglets.
(DOC)

S2 Text The cluster of differentially expressed proteins. (XLSX)

## References

[1] Moeser AJ, Klok CV, Ryan KA, Wooten JG, Little D, Cook VL, et al (2007) Stress signaling pathways activated by weaning mediate intestinal dysfunction in the pig. Am J Physiol Gastrointest Liver Physiol 292: G173–G181. 16901995 10.1152/ajpgi.00197.2006

[2] van Beers-Schreurs HMG, Nabuurs MJ, Vellenga L, Kalsbeek-van der Valk HJ, Wensing T, Breukink HJ (1998) Weaning and the weanling diet influence the villous height and crypt depth in the small intestine of pigs and alter the concentrations of short-chain fatty acids in the large intestine and blood. J Nutr 128: 947–953. 9614152 10.1093/jn/128.6.947

[3] Lallès JP, Boudry G, Favier C, Le Floc’h N, Luron I, Montagne L, et al (2004) Gut function and dysfunction in young pigs: physiology. Anim Res 53(4): 301–316.

[4] Pluske JR, Hampson DJ, Williams IH (1997) Factors influencing the structure and function of the small intestine in the weaned pig: a review. Livest Prod Sci 51: 215–236.

[5] Boudry G, Péron V, Le Huërou-Luron I, Lallès JP, Sève B (2004) Weaning induces both transient and long-lasting modifications of absorptive, secretory, and barrier properties of piglet intestine. J Nutr 134: 2256–2262. 15333713 10.1093/jn/134.9.2256

[6] Miller BG, Skadhauge E (1997) Effect of weaning in the pig on ileal ion transport measured in vitro. J Vet Med A 44:289–299. 10.1111/j.1439-0442.1997.tb01113.x 9274148

[7] Vente-Spreeuwenberg MAM, Verdonk JMA, Gaskins HR, Verstengen MWA (2001) Small intestine epithelial barrier function is compromised in pigs with low feed intake at weaning. J Nutr 131: 1520–1527. 11340110 10.1093/jn/131.5.1520

[8] Montagne L, Boudry G, Favier C, Le Huërou-Luron I, Lallès JP, Sève B (2007) Main intestinal markers associated with the changes in gut architecture and function in piglets after weaning. Br J Nutr 97(1): 45–57. 17217559 10.1017/S000711450720580X

[9] McCracken BA, Spurlock ME, Roos MA, Zuckermann FA, Gaskins HR (1999) Weaning anorexia may contribute to local inflammation in the piglet small intestine. J Nutr 129: 613–619. 10082764 10.1093/jn/129.3.613

[10] Pié S, Lalles JP, Blazy F, Laffitte J, Sève B, Oswald IP (2004) Weaning is associated with an up-regulation of expression of inflammatory cytokines in the intestine of piglets. J Nutr 134: 641–647. 14988461 10.1093/jn/134.3.641

[11] Lopez-Pedrosa JM, Torres MI, Fernandez MI, Rios A, Gill A (1998) Severe malnutrition alters lipid composition and fatty acid profile on the small intestine in newborn piglets. J Nutr 128: 224–233. 9446848 10.1093/jn/128.2.224

[12] Yang H, Xiong X, Yin Y (2013) Development and renewal of intestinal villi in pigs In: Blachier F, Wu G, Yin Y, editors. Nutritional and Physiological Functions of Amino Acids in Pigs. New York: Springer pp. 29–47.

[13] Pinto D, Clevers H (2005) Wnt control of stem cells and differentiation in the intestinal epithelium. Exp Cell Res 306(2): 357–363. 15925592 10.1016/j.yexcr.2005.02.022

[14] Fan MZ, Stoll B, Jiang R, Burrin DG (2001) Enterocyte digestive enzyme activity along the crypt-villus and longitudinal axes in the neonatal pig small intestine. J Anim Sci 79: 371–381. 11219446 10.2527/2001.792371x

[15] Fan MZ, Matthews JC, Etienne NM, Stoll B, Lackeyram D, Burrin DG (2004) Expression of apical membrane L-glutamate transporters in neonatal porcine epithelial cells along the small intestinal crypt-villus axis. Am J Physiol Gastrointest Liver Physiol 287: G385–398. 15044176 10.1152/ajpgi.00232.2003

[16] Yang C (2011) Expression of porcine intestinal nutrient transporters along crypt-villus axis and during postnatal development. Doctoral Thesis, University of Guelph. Available: https://dspace.lib.uoguelph.ca/xmlui/handle/10214/2584.

[17] Wiese S, Reidegeld KA, Meyer HE, Warscheid B (2007) Protein labeling by iTRAQ: a new tool for quantitative mass spectrometry in proteome research. Proteomics 7: 340–350. 17177251 10.1002/pmic.200600422

[18] Ralhan R, DeSouza LV, Matta A, Tripathi SC, Ghanny S, Datta Gupta S, et al (2008) Discovery and verification of head-and-neck cancer biomarkers by differential protein expression analysis using iTRAQ labeling, multidimensional liquid chromatography, and tandem mass spectrometry. Mol Cell Proteomics 7: 1162–1173. 10.1074/mcp.M700500-MCP200 18339795 PMC2424195

[19] National Research Council (1998) Nutrient Requirements of Swine (9th ed). Washington, DC: National Academic Press.

[20] Xiong X, Yang H, Tan B, Yang C, Wu M, Liu G, et al (2015) Differential expression of proteins involved in energy production along the crypt-villus axis in early-weaning pig small intestine. Am J Physiol Gastrointest Liver Physiol 309(4): G229–G237. 10.1152/ajpgi.00095.2015 26045611

[21] Conesa A, Götz S, García-Gómez JM, Terol J, Talón M, Robles M (2005) Blast2GO: a universal tool for annotation, visualization and analysis in functional genomics research. Bioinformatics 21(18): 3674–3676. 16081474 10.1093/bioinformatics/bti610

[22] KEGG (2014) Available: http://www.genome.jp/kegg/.

[23] Ye J, Fang L, Zheng H, Zhang Y, Chen J, Zhang Z, et al (2006) WEGO: a web tool for plotting GO annotations. Nucleic Acids Res 34: W293–297. 16845012 10.1093/nar/gkl031 PMC1538768

[24] de Hoon MJL, Imoto S, Nolan J, Miyano S (2004) Open source clustering software. Bioinformatics 20(9): 1453–1454. 14871861 10.1093/bioinformatics/bth078

[25] Yang H, Li F, Kong X, Yuan X, Lian G, Geng M, et al (2012) Molecular cloning, tissue distribution and ontogenetic expression of Xiang pig chemerin and its involvement in regulating energy metabolism through Akt and ERK1/2 signaling pathways. Mol Biol Rep 39(2): 1887–1894. 10.1007/s11033-011-0934-8 21643960

[26] Yang HS, Fu DZ, Kong XF, Wang WC, Yang XJ, Nyachoti CM, et al (2013) Dietary supplementation with N-carbamylglutamate increases the expression of intestinal amino acid transporters in weaned Huanjiang mini-pig piglets. J Anim Sci 91(6): 2740–2748. 10.2527/jas.2012-5795 23478823

[27] Moeser AJ, Ryan KA, Nighot PK, Blikslager AT (2007) Gastrointestinal dysfunction induced by early weaning is attenuated by delayed weaning and mast cell blockade in pigs. Am J Physiol Gastrointest Liver Physiol 293(2): G413–G421. 17525151 10.1152/ajpgi.00304.2006

[28] Gu X, Li D, She R (2002) Effect of weaning on small intestinal structure and function in the piglet. Arch Anim Nutr 56(4): 275–286. 10.1080/00039420214345 12462912

[29] Verdonk J, Bruininx E, Van Der Meulen J, Verstegen MWA (2007) Post-weaning feed intake level modulates gut morphology but not gut permeability in weaned piglets. Livest Sci 108(1): 146–149.

[30] de Roos B, McArdle HJ (2008) Proteomics as a tool for the modelling of biological processes and biomarker development in nutrition research. Brit J Nutr 99(S3): S66–S71. 18598591 10.1017/S0007114508006909

[31] Zhu LH, Xu JX, Zhu SW, Cai X, Yang SF, Chen XL, et al (2014) Gene expression profiling analysis reveals weaning-induced cell cycle arrest and apoptosis in the small intestine of pigs. J Anim Sci 92: 996–1006. 10.2527/jas.2013-7551 24496830

[32] Hansson J, Panchaud A, Favre L, Bosco N, Mansourian R, Benyacoub J, et al (2011) Time-resolved quantitative proteome analysis of in vivo intestinal development. Mol Cell Proteomics 10: M110.005231. 10.1074/mcp.M110.005231 PMC3047163 21191033

[33] Brooks PH, Moran CA, Beal JD, Demeckova V, Campbell A (2001) Liquid feeding for the young piglet In: Varley MA, Wiseman J, editors. The weaner pig: nutrition and management. Wallingford: CAB International pp. 153–178.

[34] Heo JM, Opapeju FO, Pluske JR, Kim JC, Hampson DJ, Nyachoti CM (2013) Gastrointestinal health and function in weaned pigs: a review of feeding strategies to control post-weaning diarrhoea without using in-feed antimicrobial compounds. J Anim Physiol and AN N 97: 207–237. 10.1111/j.1439-0396.2012.01284.x 22416941

[35] Van Der Schoor SR, Reeds PJ, Stoll B, Henry JF, Rosenberger JR, Burrin DG, et al (2002) The high metabolic cost of a functional gut. Gastroenterology 123: 1931–1940. 12454850 10.1053/gast.2002.37062

[36] van Goudoever JB, Stoll B, Henry JF, Burrin DG, Reeds PJ (2000) Adaptive regulation of intestinal lysine metabolism. Proc Natl Acad Sci USA 97: 11620–11625. 11016965 10.1073/pnas.200371497 PMC17250

[37] Ricquier D (2005) Respiration uncoupling and metabolism in the control of energy expenditure. P Nutr Soc 64(01): 47–52. 10.1079/pns2004408 15877922

[38] Alpers DH (1972) Protein synthesis in intestinal mucosa: the effect of route of administration of precursor amino acids. J Clin Invest 51(1): 167–173. 5007047 10.1172/JCI106788 PMC332942

[39] Chang J, Chance MR, Nicholas C, Ahmed N, Guilmeau S, Flandez M, et al (2008) Proteomic changes during intestinal cell maturation in vivo. J Proteomics 71: 530–546. 10.1016/j.jprot.2008.08.003 18824147 PMC2655360

[40] Fraser CS (2009) The molecular basis of translational control. Prog Mol Biol Transl Sci 90: 1–51. 10.1016/S1877-1173(09)90001-1 20374738

[41] Mahoney SJ, Dempsey JM, Blenis J (2009) Cell signaling in protein synthesis: ribosome biogenesis and translation initiation and elongation. Prog Mol Biol Transl Sci 90: 53–107. 10.1016/S1877-1173(09)90002-3 20374739

[42] Sonenberg N, Hinnebusch AG (2009) Regulation of translation initiation in eukaryotes: mechanisms and biological targets. Cell 136: 731–745. 10.1016/j.cell.2009.01.042 19239892 PMC3610329

[43] Sengupta S, Peterson TR, Sabatini DM (2010) Regulation of the mTOR complex 1 pathway by nutrients, growth factors, and stress. Mol Cell 40: 310–322. 10.1016/j.molcel.2010.09.026 20965424 PMC2993060

[44] Deplancke B, Gaskins HR (2001) Microbial modulation of innate defense: goblet cells and the intestinal mucus layer. Am J Clin Nutr 73(6): 1131S–1141S. 11393191 10.1093/ajcn/73.6.1131S

[45] Perez-Vilar J, Hill RL (1999) The structure and assembly of secreted mucins. J Biol Chem 274(45): 31751–31754. 10542193 10.1074/jbc.274.45.31751

[46] Kim YS, Ho SB (2010) Intestinal goblet cells and mucins in health and disease: recent insights and progress. Curr Gastr Rep 12(5): 319–330. 10.1007/s11894-010-0131-2 PMC2933006 20703838

[47] Forstner JF, Oliver MG, Sylvester FA (1995) Production, structure, and biologic relevance of gastrointestinal mucins In: Blaser MJ, Smith PD, Ravdin JI, Greenberg HB, Guerrant RL, editors. Infections of the gastrointestinal tract. New York: Raven Press pp. 71–88.

[48] Mackie AR, Round AN, Rigby NM, Macierzanka A (2012) The role of the mucus barrier in digestion. Food Digestion 3: 8–15.

[49] Wang J, Chen L, Li P, Li X, Zhou H, Wang F, et al (2008) Gene expression is altered in piglet small intestine by weaning and dietary glutamine supplementation. J Nutr 138(6): 1025–1032. 18492829 10.1093/jn/138.6.1025

[50] Liu Y, Huang J, Hou Y, Zhu H, Zhao S, Ding B, et al (2008) Dietary arginine supplementation alleviates intestinal mucosal disruption induced by Escherichia coli lipopolysaccharide in weaned pigs. Br J Nutr 100: 552–560. 10.1017/S0007114508911612 18275628

[51] Stappenbeck TS, Gordon JI (2000) Rac1 mutations produce aberrant epithelial differentiation in the developing and adult mouse small intestine. Development 127(12): 2629–2642. 10821761 10.1242/dev.127.12.2629

