# Supplementary material for: Effects of Weaning on Intestinal Upper Villus Epithelial Cells of Piglets
Source: PLoS One. 2016 Mar 29;11(3):e0150216. doi: 10.1371/journal.pone.0150216 (PMC4811545; doi:10.1371/journal.pone.0150216)
Supplement: S1 Text — Table A. Primers used for PCR analysis. Fig A Cellular component, molecular function, and biological process ontology of differentially expressed proteins in jejunal upper villus epithelial cells of piglets during the post-weaning period. Fig B KEGG pathways enrichment of differentially expressed proteins in jejunal upper villus epithelial cells of weaning piglets. Fig C Effects of weaning on the expression of proteins in mammalian target of rapamycin signaling pathway (mTOR) in jejunal upper villus epithelial cells of piglets. The expression of proteins in mTOR signaling pathway was measured using Western blotting. Fig D Effect of weaning on the expression of I-FABP, Bcl-2, and caspase-3 in jejunal upper villus epithelial cells of piglets. (DOC) [file pone.0150216.s001.doc]

**Table A.** Primers used for PCR analysis

| Genes | Sequences（50–30） | Genebank accession | Product size |
| --- | --- | --- | --- |
| *PYK* | TCGCATCTTTCATCCGTAA | XM_005666189.1 | 124 bp |
|  | TCATCAAATCTCCGAACTCC |  |  |
| *CISN* | ATGAAGGTGGCAATGTAAG | NM_214276.1 | 228 bp |
|  | CCCGTCCTGAGTTGAGTG |  |  |
| *ICDH* | ATTCTGAAAGCCTACGACG | NM_001164007.1 | 143 bp |
|  | GAAGACTTGAGGACCTGAGC |  |  |
| *OxoGDH* | CGTGACCGACAGGAACATC | XM_003134891.4 | 239 bp |
|  | CGTGGACAGTGCCGTGAG |  |  |
| *CPT1* | GTGTCGCCAAGCCTATTT | NM_001129805.1 | 224 bp |
|  | GGAGTGCTCAGCGTTCAT |  |  |
| *CPT2* | GCTTTGGCATTGGGTAT | NM_001246243.1 | 64 bp |
|  | CTTTGGTAGGCGGAGAC |  |  |
| *L-ACD* | GAGTAAGAACAAATGCCAAGA | NM_213897.1 | 105 bp |
|  | CAGCCACTACAATCACAACA |  |  |
| *ACO* | TATGCCTTCCAGTTTGTTG | NM_001101028.1 | 52 bp |
|  | TAATGCGGTGATAGGTCTCT |  |  |
| *β-actin* | TGCGGGACATCAAGGAGAAG | XM_003357928.2 | 216 bp |
|  | AGTTGAAGGTGGTCTCGTGG |  |  |

*PYK*, Pyruvate kinase; *CISN*, Citrate synthase; *ICDH*, isocitrate dehydrogenase; *OxoGDH*, Oxoglutarate dehydrogenase; *CPT1*, Carnitine palmitoyltransferase 1; *CPT2*, Carnitine palmitoyltransferase 2; *L-ACD*, Long-chain acyl-CoA dehydrogenase; *ACO*, acyl-CoA oxidase.

**Fig. A.** Cellular component, molecular function, and biological process ontology of differentially expressed proteins in jejunal upper villus epithelial cells of piglets during the post-weaning period. A protein with ≥ 1.2-fold or ≤ 0.8-fold difference between W1d, W3d, W5d or W7d and W0d and a *P*-value ≤ 0.05 was regarded as being differentially expressed. The differentially expressed proteins were enriched to different GO terms using WEGO program.

**
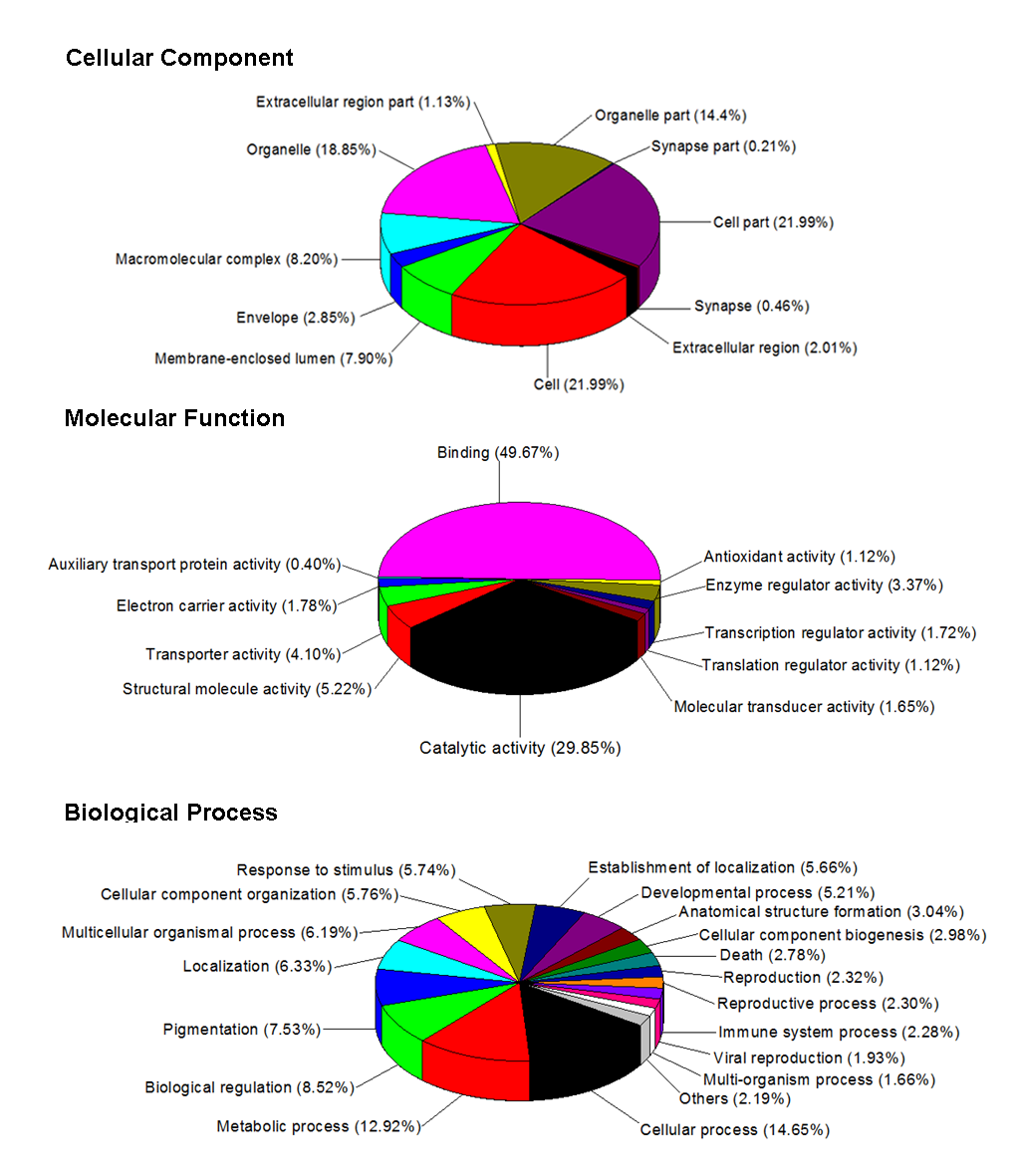
**

**Fig. B.** KEGG pathways enrichment of differentially expressed proteins in jejunal upper villus epithelial cells of weaning piglets. A protein with ≥ 1.2-fold or ≤ 0.8-fold difference in W1d, W3d, W5d or W7d compared with W0d and a *P*-value ≤ 0.05 was regarded as being differentially expressed.

**
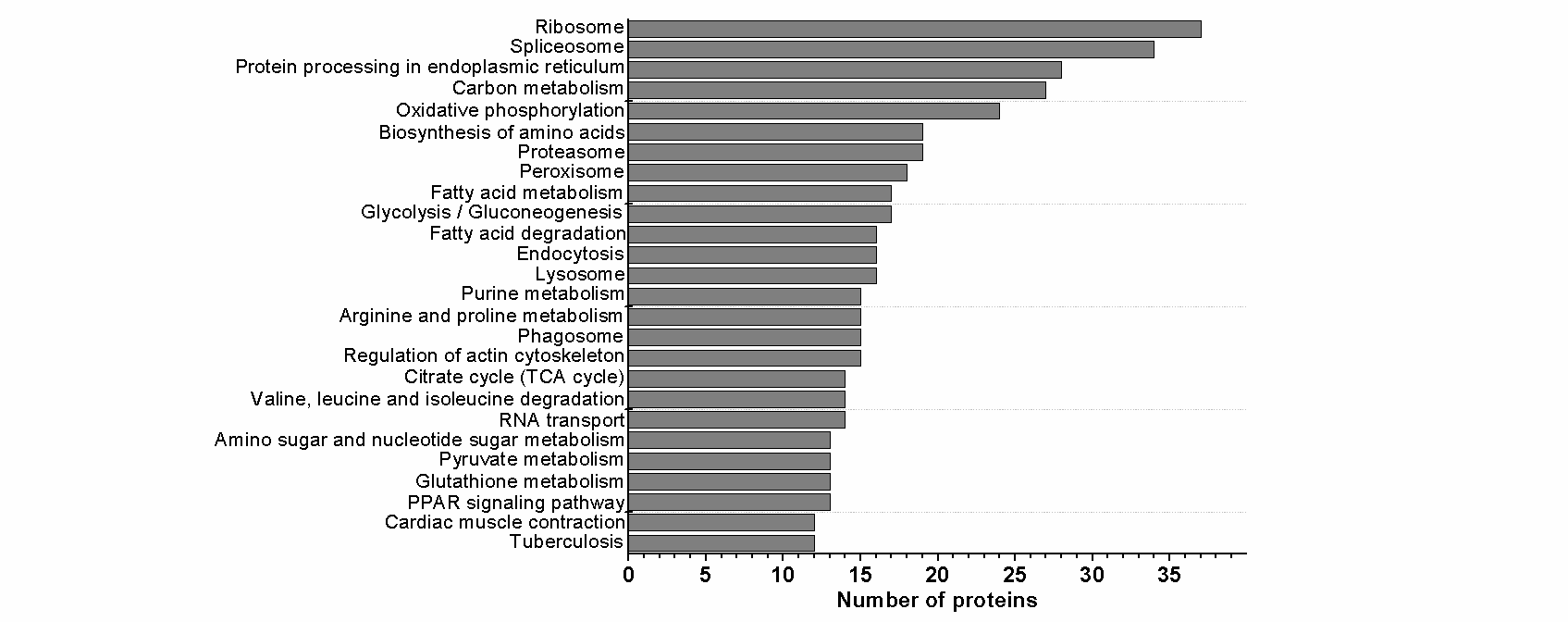
**

**Fig. C.** Effects of weaning on the expression of proteins in mammalian target of rapamycin signaling pathway (mTOR) in jejunal upper villus epithelial cells of piglets. The expression of proteins in mTOR signaling pathway was measured using Western blotting. 4EBP1, eukaryotic initiation factor-4E binding protein-1; p-4EBP1, phospho-4EBP1; p-mTOR, phospho-mTOR; S6K, S6 kinase; p-S6K, phospho-S6K; eIF4E, total eukaryotic initiation factor-4E.


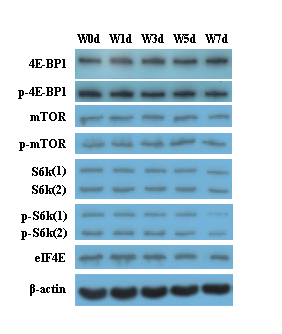


**Fig. D.** Effect of weaning on the expression of I-FABP, Bcl-2, and caspase-3 in jejunal upper villus epithelial cells of piglets. The expression of I-FABP, Bcl-2, and caspase-3 was measured using Western blotting. I-FABP, intestinal fatty aid binding protein.

*
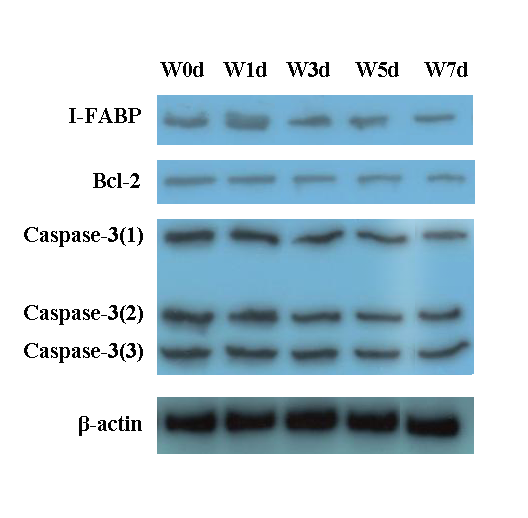
*
